# Effects of life history strategies and habitats on limb regeneration in plethodontid salamanders

**DOI:** 10.1002/dvdy.742

**Published:** 2024-09-20

**Authors:** Vivien Bothe, Hendrik Müller, Neil Shubin, Nadia Fröbisch

**Affiliations:** ^1^ Museum für Naturkunde Berlin Leibniz Institute for Evolution and Biodiversity Science Berlin Germany; ^2^ Department of Biology Humboldt University Berlin Berlin Germany; ^3^ Zentralmagazin Naturwissenschaftlicher Sammlungen Martin‐Luther‐Universität Halle‐Wittenberg Halle (Saale) Germany; ^4^ Department of Organismal Biology & Anatomy The University of Chicago Chicago Illinois USA

**Keywords:** appendage regeneration, conspecific biting, Plethodontidae, salamander, skeletal anomalies

## Abstract

**Background:**

Salamanders are the only tetrapods that exhibit the ability to fully regenerate limbs. The axolotl, a neotenic salamander, has become the model organism for regeneration research. Great advances have been made providing a detailed understanding of the morphological and molecular processes involved in limb regeneration. However, it remains largely unknown how limb regeneration varies across salamanders and how factors like variable life histories, ecologies, and limb functions have influenced and shaped regenerative capacities throughout evolution.

**Results:**

This study focuses on six species of plethodontid salamanders representing distinct life histories and habitats. Specimens were examined for regeneration ability after bite injuries as well as after controlled amputations. Morphological investigations revealed great regenerative abilities in all investigated species and frequent anatomical limb anomalies. Correlations were observed with respect to speed of regeneration and habitat.

**Conclusions:**

Investigating regeneration in non‐model salamander taxa is essential for disentangling shared features of the regeneration process versus those that may be more taxon‐specific. Gaining insights into variable aspects of regeneration under natural conditions and after conspecific biting rather than controlled amputations adds important new datapoints for understanding the evolutionary framework of regeneration and provides a broader context for interpreting findings made in the model organism axolotl.

## Key Findings

- Morphological investigations in six species of plethodontid salamanders representing distinct life histories and habitats revealed great regenerative abilities in all of the investigated species, but also frequent anatomical limb anomalies
- Correlations were observed with respect to speed of regeneration and habitat
- Investigating regeneration in non‐model salamander taxa is essential for disentangling shared features of the regeneration process versus those that may be taxon‐specific
- Gaining insights into variable aspects of regeneration under natural conditions and after conspecific biting rather than controlled amputations can add important new datapoints for understanding the evolutionary framework of regeneration and provide a broader context for interpreting findings made in the model organism axolotl

## 1 INTRODUCTION

**FIGURE 1:**
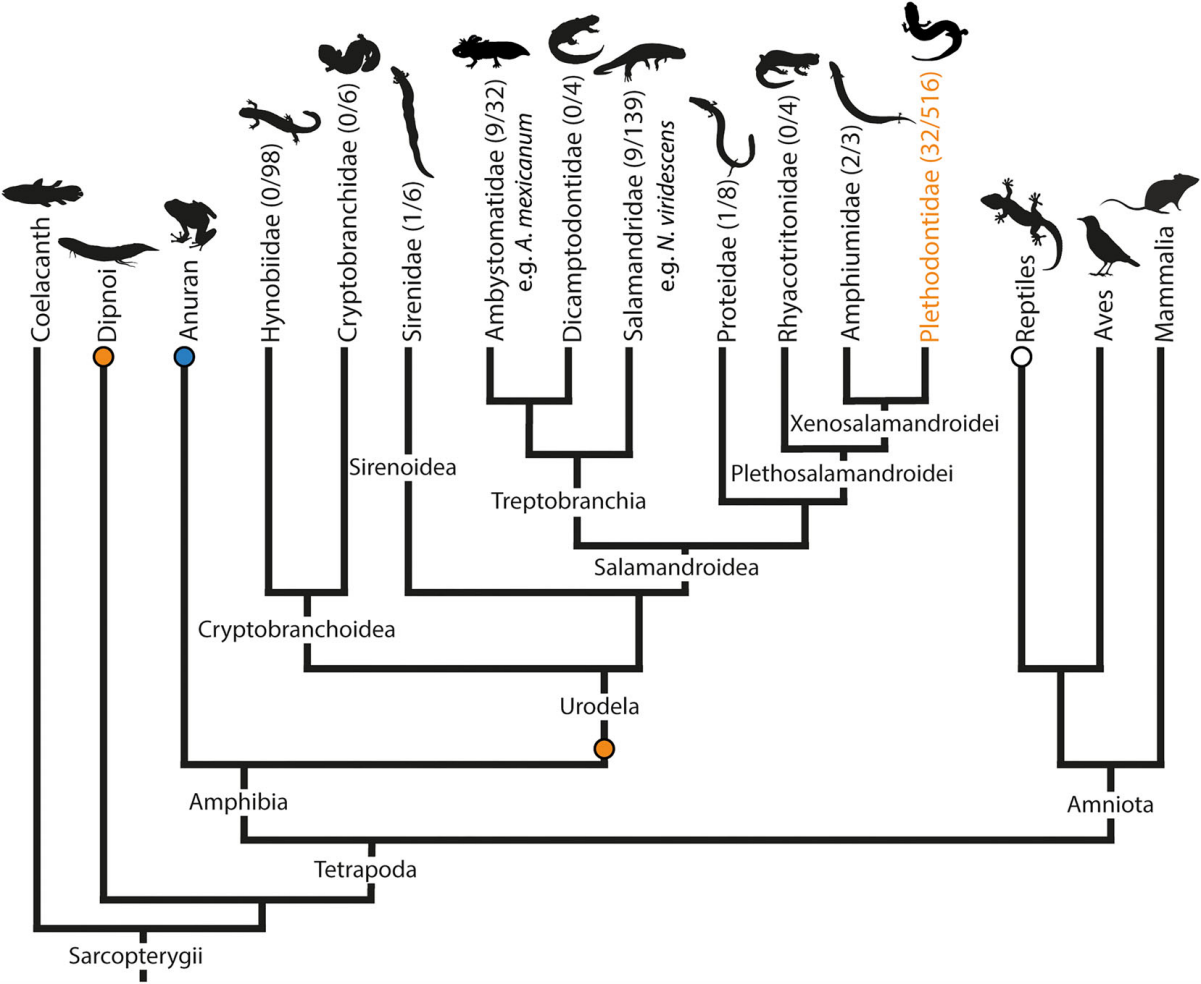
Ability of epimorphic appendage regeneration in sarcopterygians. Lineages with one or more regeneration‐competent species are indicated with an orange circle. Blue circle point to regeneration capacities of frogs and toads restricted to the larval stage. Appearance of incomplete tail regeneration in squamate amniotes (lacertid lizards and geckos) is denoted with a white circle. Numbers in brackets of Urodela families indicate species examined for regeneration (amphibiaweb.org) and total number of species within each family (amphibianweb.org, as of Mai 2024). Phylogeny modified after Pyron and Wiens (2011).

Among modern four‐legged vertebrates (tetrapods) epimorphic regeneration is very rare.^1^, ^2^ Indeed, salamanders are the only tetrapods able to regenerate various anatomical structures, including organs such as liver, heart, and lens as well as body appendages (limbs and tails) repeatedly and throughout their entire life span^3^, ^4^, ^5^, ^6^ (Figure 1). While some studies have suggested that certain salamanders may not be able to regenerate their limbs at all,^7^ currently available data suggest that most or even all salamander species are capable of regeneration.^8^, ^9^, ^10^, ^11^, ^12^

Among amniotes, squamates (especially lacertid lizards and geckos) are well‐known for their ability to regenerate tails as decoy against predators. However, contrary to the tail regeneration in salamanders, which results in a fully functional tail including vertebrae and neural spine, squamate tail regeneration only yields an unsegmented hollow cartilage tube instead of segmented tail vertebrae^13^, ^14^ and the regenerated muscles and skin display abnormal structures.^15^, ^16^, ^17^, ^18^, ^19^ Although mammals show even less regenerative capacity and tissue repair usually leads to non‐functional fibrotic tissue formation,^3^, ^20^ human fingertips are capable of a regenerative response distal to the nail bed.^21^, ^22^, ^23^

Especially, the capacity of salamanders to regenerate limbs has generated extensive research interest over the past decades.^11^, ^24^, ^25^, ^26^, ^27^ Limb regeneration is a remarkably coordinated process that generally results in a new, fully functional extremity that cannot easily be differentiated from a non‐regenerated limb. Particularly, the initial steps of the regeneration process, wound healing and blastema formation, are well understood by now and significant advances have been made in revealing the molecular pathways involved in limb regeneration.^28^, ^29^, ^30^

The remarkable advances in research on tetrapod regeneration have largely been based on a small number of salamander species, first and foremost the Mexican axolotl *Ambystoma mexicanum* as well as to a lesser extent *Pleurodeles waltl* and *Notophthalmus viridescens*. The axolotl will undoubtedly continue to be a crucial taxon for this field of research and the future of regenerative medicine.^31^, ^32^ However, as for any model organism, it is important to keep in mind that the axolotl is a species with its own biological specifications, which set limits to extrapolation and generalization of observed patterns to larger phylogenetic clades. Salamanders are a highly diverse clade of tetrapods with around 850 currently known species in 10 major clades (amphibiaweb.org) and an evolutionary history that reaches back at least into the late Triassic period, some 230 million years ago.^33^ Therein, the axolotl, *A. mexicanum*, belongs to the Ambystomatidae, a comparatively derived clade of urodeles.^34^ The clade contains species with varying life history pathways including metamorphosis into a terrestrial adult after an aquatic larval stage, neoteny, a life history pattern forgoing metamorphosis entirely, and species that are facultatively neotenic, where an individual's life history pattern depends on complex ecological cues.^35^ The axolotl is an aquatic, obligatorily neotenic species that retains larval somatic features like external gills, an overall weakly ossified skeleton, and a laterally compressed tail with broad tailfins for aquatic locomotion into adult stages.^36^, ^37^ In their physiology, morphology, and the somatic maturity of certain organs, adult axolotls are therefore more similar to larval salamanders than metamorphosed adults. It is conceivable that this may indeed play an important role in its regeneration program, an assumption that is supported by data showing that speed and quality of regeneration in amphibians decrease abruptly after metamorphosis.^7^, ^38^, ^39^ Limb regeneration has also been demonstrated to be slower in adult axolotls than in larvae^40^ and regenerates of sexually mature individuals show more pathologies than in larvae,^41^ even more so, when metamorphosis was induced artificially.^42^ These data point to a greater variability in the regeneration process than currently known and appreciated. Regeneration research has now achieved a level of knowledge that allows for, and requires, the addition of a broader taxonomic and evolutionary viewpoint, in order to establish common versus variable patterns of regeneration in salamanders and understand the roles life history, ecology, and functional demands may play. Thus far knowledge on regeneration in non‐model salamander species remains comparatively scarce, especially when considering the great taxonomic and ecological diversity of the clade (Figure 1). Several other *Ambystoma* species have been the subject of regeneration studies.^43^ Among them is the tiger salamander *A. tigrinum*, which in contrast to the axolotl, undergoes metamorphosis into a fully terrestrial, air‐breathing adult.^44^, ^45^, ^46^, ^47^, ^48^, ^49^, ^50^ While the tiger salamander generally shows similar regenerative abilities to the axolotl, some notable differences throughout the ontogeny of this species were documented during tail regeneration, that seem to be associated with metamorphosis.^51^, ^52^ Similarly, Tanaka et al.^53^ demonstrated cellular differences in muscle regeneration between premetamorphic and postmetamorphic stages of the aquatic salamandrid *N. viridescens*. In this species, larval individuals used progenitor cells such as satellite cells for muscle restoration comparable to axolotl, while metamorphs relied on dedifferentiation of muscle fibers. *Notophthalmus viridescens* was also used in other regeneration studies^54^, ^55^, ^56^, ^57^, ^58^ in addition to further species of the Salamandridae, such as *Pleurodeles waltl* ^59^, ^60^, ^61^, ^62^ and *Triturus cristatus*,^63^, ^64^, ^65^, ^66^ although these studies did not take a strictly comparative approach to data gained from axolotl. Data on regeneration in salamanders outside of ambystomatids and salamandrids are even scarcer^43^ and a comprehensive picture of regeneration in different salamander clades is currently lacking. However, the data at hand suggest that regeneration indeed varies between different clades both on the morphological and molecular level.^55^, ^59^, ^67^

In this study, we seek to gain a better understanding of the speed and accuracy of regeneration as well as appearance and form of pathologies in non‐model, wild salamander taxa representing a range of habitats, locomotory demands, and life history patterns. For this, we investigated limb regeneration in the highly derived and most speciose salamander clade Plethodontidae.^68^, ^69^, ^70^ Six plethodontid species were collected in the wild and limb regeneration was investigated in a comparative study after naturally induced injuries, as well as after controlled amputations along several positions along the limb axis.

## 2 RESULTS

In this study, skeletal elements of regenerated and non‐regenerated limbs of six plethodontid species were analyzed in adult individuals. Albeit all members of the clade Plethodontidae, the investigated taxa were chosen because they represent different ecologies, body sizes, and life history strategies (Table 1).

**TABLE 1:** Overview of the plethodontid species used in this study, with information on habitat and life history pattern.

Genus	Species	Habitat	Life history pattern
*Desmognathus*			
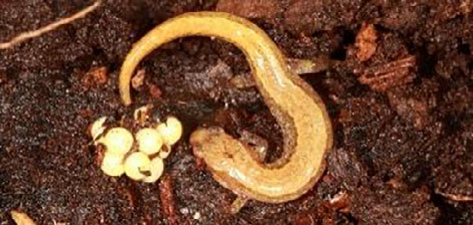	*D. aeneus* (Seepage salamander)	Seepage areas in forests	Direct development
		Leaf litter (terrestrial)	
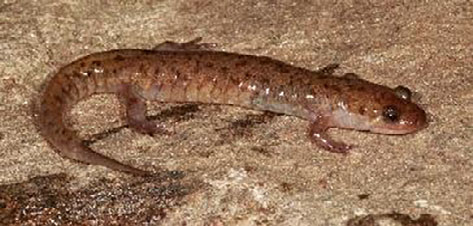	*D. monticola* (Seal salamander)	Rocky mountain streams	Larval stage
		Under rocks or moss	
		On wet rock faces (climbing)	
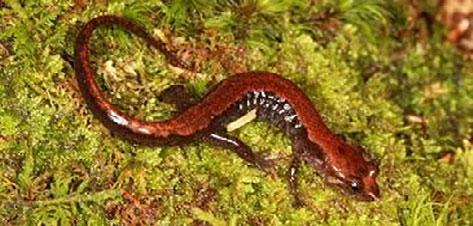	*D. ocoee* (Ocoee salamander)	Close to streams	Larval stage
		On moist forest floors	
		Wet rock faces (climbing)	
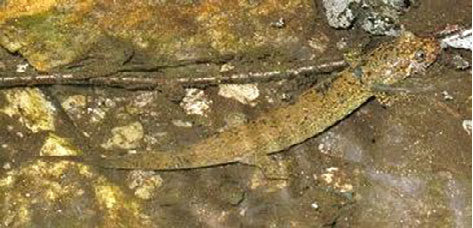	*D. quadramaculatus* (Blackbelly salamander)	Cold mountain streams	Larval stage
		Under rocks (largely aquatic)	
*Plethodon*			
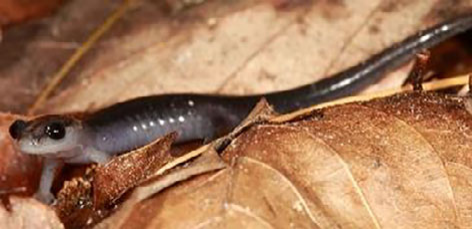	*P. metcalfi* (Southern gray‐cheeked salamander)	Under logs, mossy rocks by day	Direct development
		On the forest floor at night (terrestrial)	
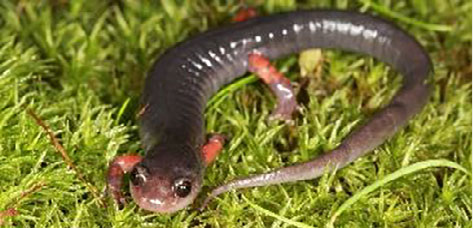	*P. shermani* (Red‐legged salamander)	Under logs, mossy rocks by day	Direct development
		On the forest floor at night (terrestrial)	

A summary of all anomalies observed in the limb anatomy, both after amputation and caused by natural regenerative processes, can be found in Table 2.

**TABLE 2:** Overview of identified anomalies in regenerated limbs of plethodontid salamander following amputation/probably regenerated limbs after injuries caused by natural factors such as conspecifics or other predatory salamander species.

	Extra carpal/tarsal	Missing or fused carpal/tarsal	Extra digit/toe	Missing digit/toe	Missing phalange	Bulky bone zeugopod	Bulky bone stylopod	Bulky bone digits	Fracture long bone
*D. aeneus* (*n* = 4)	1/0	—	1/0	—	1/0	2/0	2/0	—	0/1
*D. monticola* (*n* = 7/2*)	1/0	1/0	—	1/1	1/0	1/0	2/0	—	—
*D. ocoee* (*n* = 15)	2/0	2/0	—	1/0	2/3	8/0	2/0	0/1	0/7
*D. quadramaculatus* (*n* = 8/3*)	—	1/0	1/0		0/1	2/0	2/0	—	—
*P. metcalfi* (*n* = 8/5*)	1/0	—	—	1/0	—	—	—	—	—
*P. shermani* (*n* = 8/4*)	—	—	—	—	—	1/0	2/0	1/0	—

* indicates the number of animals that did not survive the entire duration of this study

### 2.1 Fore limb regeneration after controlled amputations

#### 2.1.1 Differences in forelimb regeneration time and external observations

**FIGURE 2:**
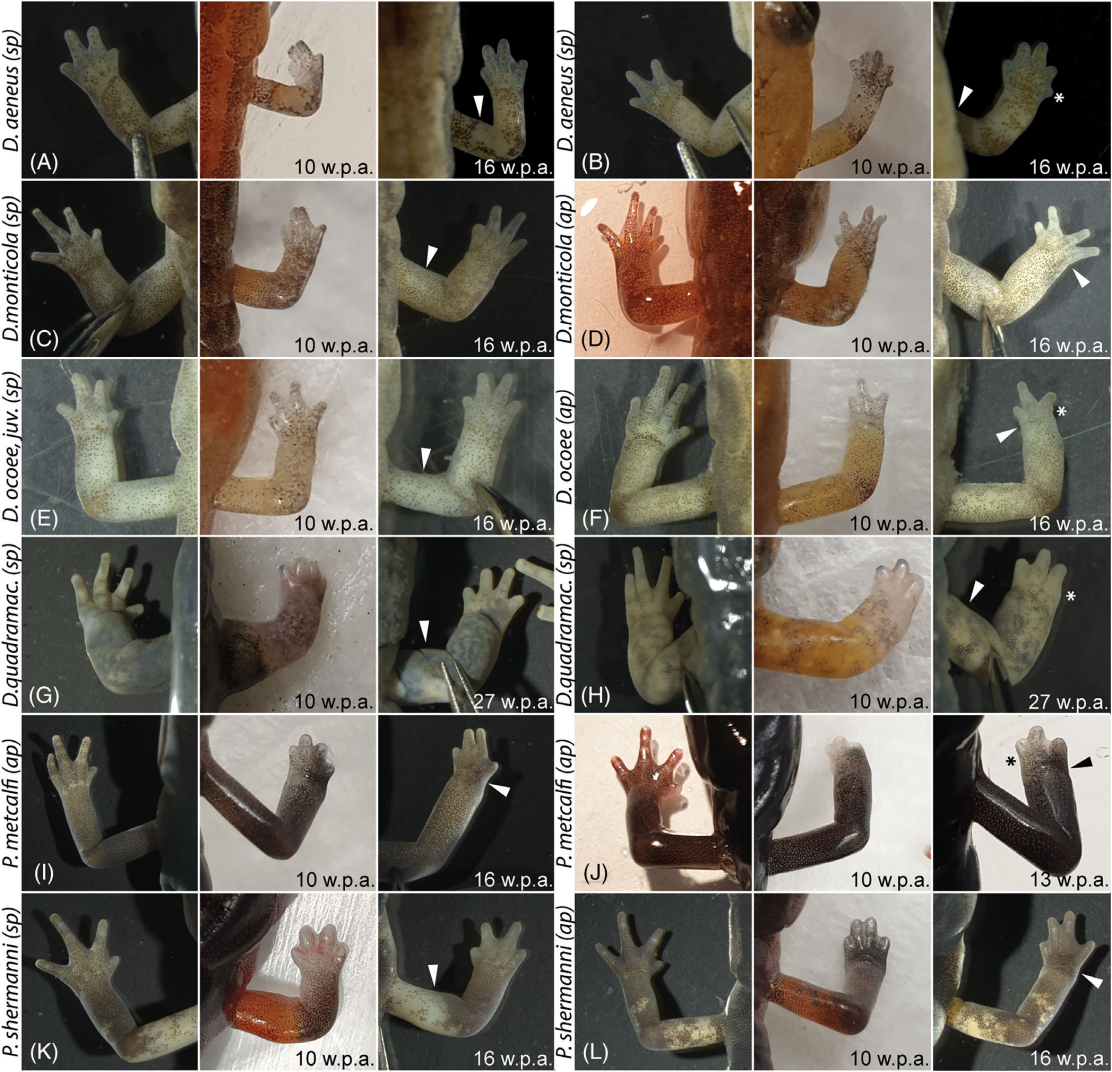
In‐lab amputations and outcomes of long‐term fore limb regeneration. *Desmognathus aeneus* with normal regenerated limb (A), regenerate with extra digit (B). *Desmognathus monticola* with normal regenerated limb (C), regenerate with conspicuous spreading of digits (D). *Desmognathus ocoee* with normal regenerated limb (E), regenerate with missing digit (F). *Desmognathus quadramaculatus* with normal regenerated limb (G), regenerate with missing digit (H). *Plethodon metcalfi* with normal regenerated limb (I), regenerate with missing digit (J). *Plethodon shermani* with normal regenerated limb (K), regenerate with conspicuous spreading of digits (L). White arrowheads highlight the amputation site. Asterisks indicate supernumerary or missing digits. sp, amputation site stylopod; ap, amputation site autopod; w.p.a., weeks post amputation.

Clear differences in terms of speed of the overall limb regeneration process until finalized replacement of the missing limb parts in their original size could be detected between the investigated taxa. The two rock‐surface dwellers *Desmognathus monticola* (Figure 2C) and *D. ocoee* (Figure 2E) are the fastest in limb regeneration among the investigated taxa. An almost complete length of the limb is attained after only 10 weeks post amputation at the stylopod level, though at this time individual fingers are still lengthening and differentiating. While these two species have fully completed the forelimb regeneration after about 16 weeks, the small, direct developing *D. aeneus* (Figure 2A) and the large, aquatic *D. quadramaculatus* (Figure 2G) required more time to achieve the normal limb morphology, especially with respect to the final extension of the digits. Even after 27 weeks post amputation at the stylopod level, the forelimbs of *D. quadramaculatus* were still undergoing regeneration.

Regeneration also took longer for the two *Plethodon* species compared to *D. monticola* and *D. occoe*. The autopod had regenerated after 16 weeks, but digit length was still noticeably smaller than in the original leg (Figure 2K). This was even the case in amputations performed at a distal, autopodial level through the carpal bones (Figure 2I).

Externally, the majority of the regenerated limbs appeared anatomically normal and indistinguishable from the non‐amputated limbs. Some anomalies, however, were already externally evident in a few cases (see also section on pathologies below). One individual of *D. aeneus* possessed an extra digit (Figure 2B), while one individual each of *D. ocoee* (Figure 2F), *D. quadramaculatus* (Figure 2H), and *P. metcalfi* (Figure 2J) was missing one digit. One individual of *D. monticola* (Figure 11C) was missing one toe, one individual of *D. monticola* displayed conspicuous, unnatural spreading of the digits (Figure 2D) and one individual of *P. shermani* (Figure 2L) showed a possible fusion of digits II and III. Despite these anomalies, all regenerated limbs appeared fully functional.

#### 2.1.2 Internal anatomy of regenerated forelimbs

For comparative purposes, non‐regenerated limbs were investigated in addition to regenerates to visualize normal limb skeletal anatomy and to check for any possible abnormalities, not apparent from the outside. Wild type anatomy of an adult plethodontid salamander limb includes a stylopod (humerus/femur) and zeugopod with two elements (radius and ulna/tibia and fibula) consisting of evenly shaped, slender long bones with the smallest diameter in the area of the diaphysis (green renderings in Figures 4 and 6). Distally, the autopod consists of cartilaginous carpalia/tarsalia, ossified metacarpalia/metatarsalia, and digits with the standard phalangeal formula 1‐2‐3‐2 in forelimbs or 1‐2‐3‐3‐2 in hind limbs.

**FIGURE 3:**
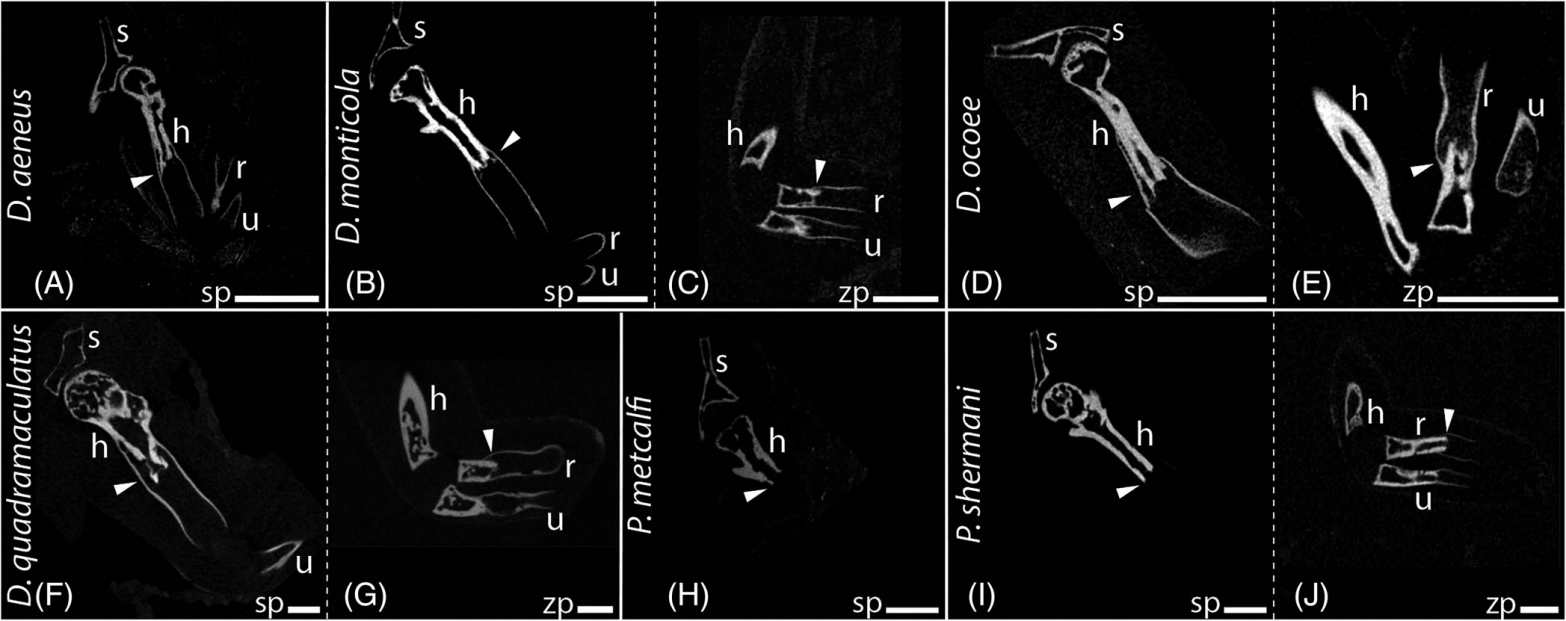
In‐lab amputations and outcomes of long‐term fore limb regeneration. Micro‐CT scans of regenerated limbs in unstained condition. *Desmognathus aeneus* (A); *D. monticola* (B‐C); *D. ocoee* (D‐E); *D. quadramaculatus* (F‐G); *Plethodon* metcalfi (H); *P. shermani* (I‐J). h, humerus; r, radius; s, scapula; u, ulna. sp, amputation site stylopod; zp, amputation site zeugopod. White arrows highlight the amputation site. White scale bars represent 1 mm.

In regenerated limbs following amputations, the level of amputation in stylopod and zeugopod, respectively, is clearly visible in the CT scans (Figure 3).

**FIGURE 4:**
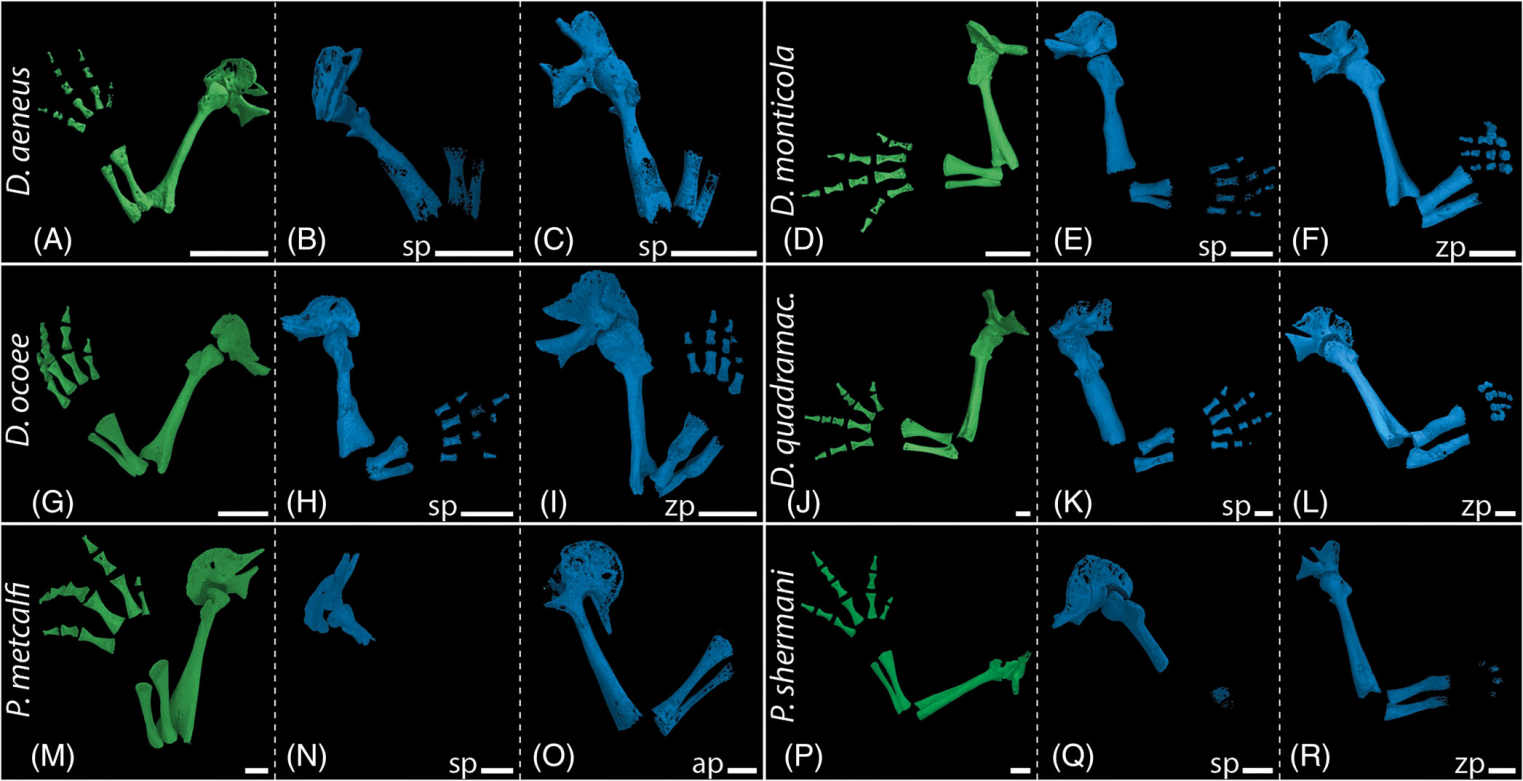
In‐lab amputations and outcomes of long‐term fore limb regeneration. Segmented 3D models of unstained micro‐CT scans showing bulky long bones after regeneration. *Desmognathus aeneus*: Representative image of a normal, non‐regenerated limb (A). Regenerated limbs after amputation at the humerus (B and C). *Desmognathus monticola*: Representative image of a normal limb (D). Regenerated limbs after amputation at the humerus (E) and radius/ulna(F). *Desmognathus ocoee*: Representative image of a normal limb (G). Regenerated limbs after amputation at the humerus (H) and radius/ulna (I). *Desmognathus quadramaculatus*: Representative image of a normal limb (J). Regenerated limbs after amputation at the humerus (K) and radius/ulna (L). *Plethodon metcalfi*: Representative image of a normal limb (M). Regenerated limbs after amputation at the humerus (N) and carpal bones (O). *Plethodon shermani*: Representative image of a normal limb (P). Regenerated limbs after amputation at the humerus (Q) and radius/ulna (R). Green color represents contralateral unamputated control limbs, blue color represents regenerated limbs. ap, amputation site autopod; sp, amputation site stylopod; zp, amputation site zeugopod. White scale bars represent 1 mm.

Limb patterning and outgrowth in the four *Desmognathus* species proceeded comparatively quickly after amputation, until the regenerated limbs reached a size comparable to the contralateral, unregenerated limb in about 16 weeks post amputation (w.p.a.). However, CT data clearly shows that ossification of the regenerated skeleton needs much longer and only commences 16 weeks post amputation in *D. monticola* and *D. ocoee* and 27 weeks post amputation in *D. quadramaculatus*. Full ossification would likely have taken many more months until completion. The most advanced ossification was observed in *D. monticola* (Figure 4E) and *D. ocoee* (Figure 4H). After stylopodial amputation, humerus, radius, and ulna, as well as all phalanges showed the onset of ossification of the cortical bones after 16 weeks. In contrast, after amputation at the same level in *D. aeneus*, no incipient ossification was evident in the metacarpal bones and phalanges after the same duration of time (Figure 4B,C). A direct comparison to *D. quadramaculatus* (Figure 4K,L) cannot be drawn here since these forelimbs had 11 more weeks to regenerate, which is why no CT data is available on the progress of ossification after 16 weeks.

In the two *Plethodon* species, ossification of the regenerated cartilaginous humerus had not begun after 16 w.p.a. (Figure 3H,I). However, at least in *P. shermani*, initial ossification can be observed in the zeugopods (Figure 4Q) after amputation at the humerus level at this time. In contrast, radius and ulna as well as some phalanges already show initial ossification 16 w.p.a. in this species following amputation in the zeugopod region (Figures 3J and 4R). *Plethodon metcalfi* on the other hand shows no ossification of the autopod yet 16 w.p.a after amputation at the zeugopod (Figure 4O).

**FIGURE 5:**
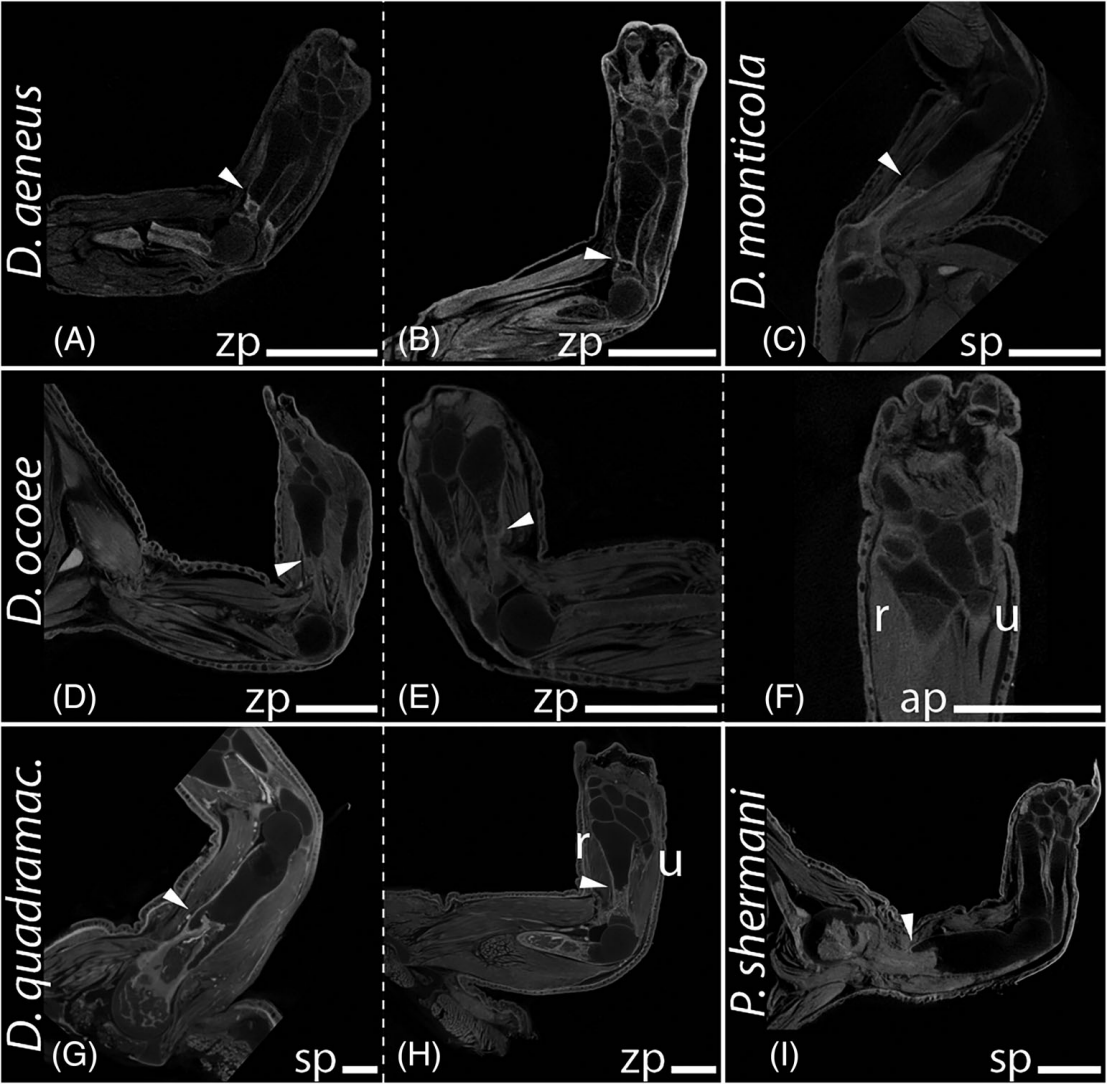
In‐lab amputations and outcomes of long‐term fore limb regeneration. Micro‐CT scans of regenerated limbs double stained with I2K and PTA. *Desmognathus aeneus*: Bulky regenerated radius and ulna (A and B). *Desmognathus monticola*: Bulky regenerated humerus (C). *Desmognathus ocoee*: Bulky regenerated radius and ulna (D and E); imperfect regeneration of the carpal bones (F). *Desmognathus quadramaculatus*: Bulky regenerated humerus (G); bulky regenerated radius (H). *Plethodon shermani*: Bulky regenerated humerus (I). ap, amputation site autopod; sp, amputation site stylopod; zp, amputation site zeugopod. White arrows highlight the amputation site. White scale bars represent 1 mm.

**FIGURE 6:**
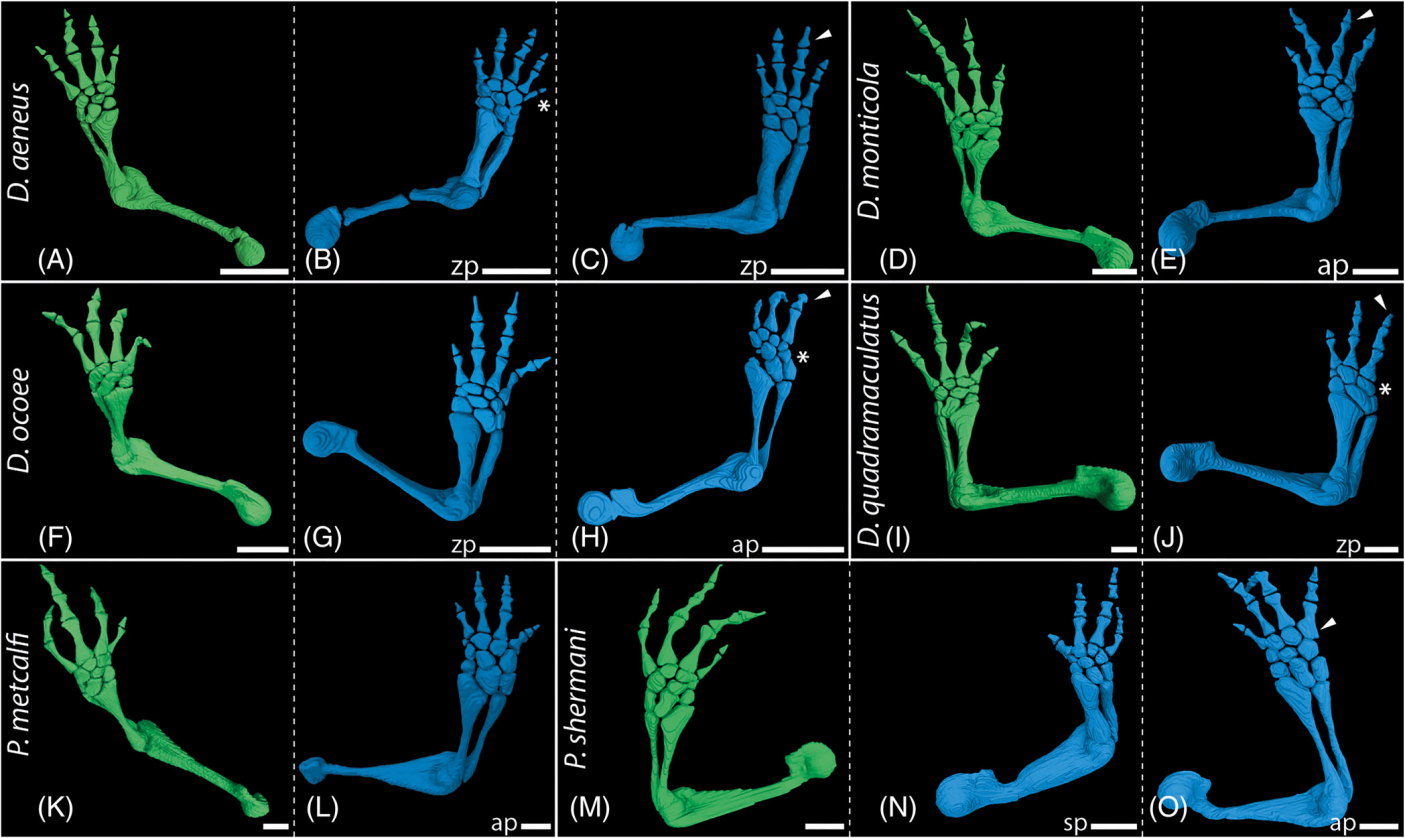
In‐lab amputations and outcomes of long‐term fore limb regeneration. Segmented 3D models of micro‐CT scans stained with I2K and PTA. Representative image of unregenerated limb anatomy of *Desmognathus aeneus* (A), *D. monticola* (D), *D. ocoee* (F), *D. quadramaculatus* (I), *Plethodon metcalfi* (K), and *P. shermani* (N). *Desmognathus aeneus* bulky radius and ulna, supernumerary digit V (B) bulky radius and ulna, missing distal phalanx on digit III (C). *Desmognathus monticola*: Missing distal phalanx on digit III (E). *Desmognathus ocoee*: Bulky radius and ulna (G); missing digit IV in whole, missing distal phalanx on digit III, deviating mesopodial anatomy (H). *Desmognathus quadramaculatus*: Missing digit IV in whole, missing distal phalanx on digit III, deviating mesopodial anatomy (J). *Plethodon metcalfi*: Deviating mesopodial anatomy (L). *Plethodon shermani*: Bulky humerus and zeugopod skeletal elements (N); bulky metacarpal in digit IV (O). Green color represents contralateral unamputated control limbs, blue color represents regenerated limbs. ap, amputation site autopod; sp, amputation site stylopod; zp, amputation site zeugopod. White arrowheads indicate missing or bulky phalangeal elements. Asterisks indicate supernumerary or missing digits. White scale bars represent 1 mm.

**FIGURE 7:**
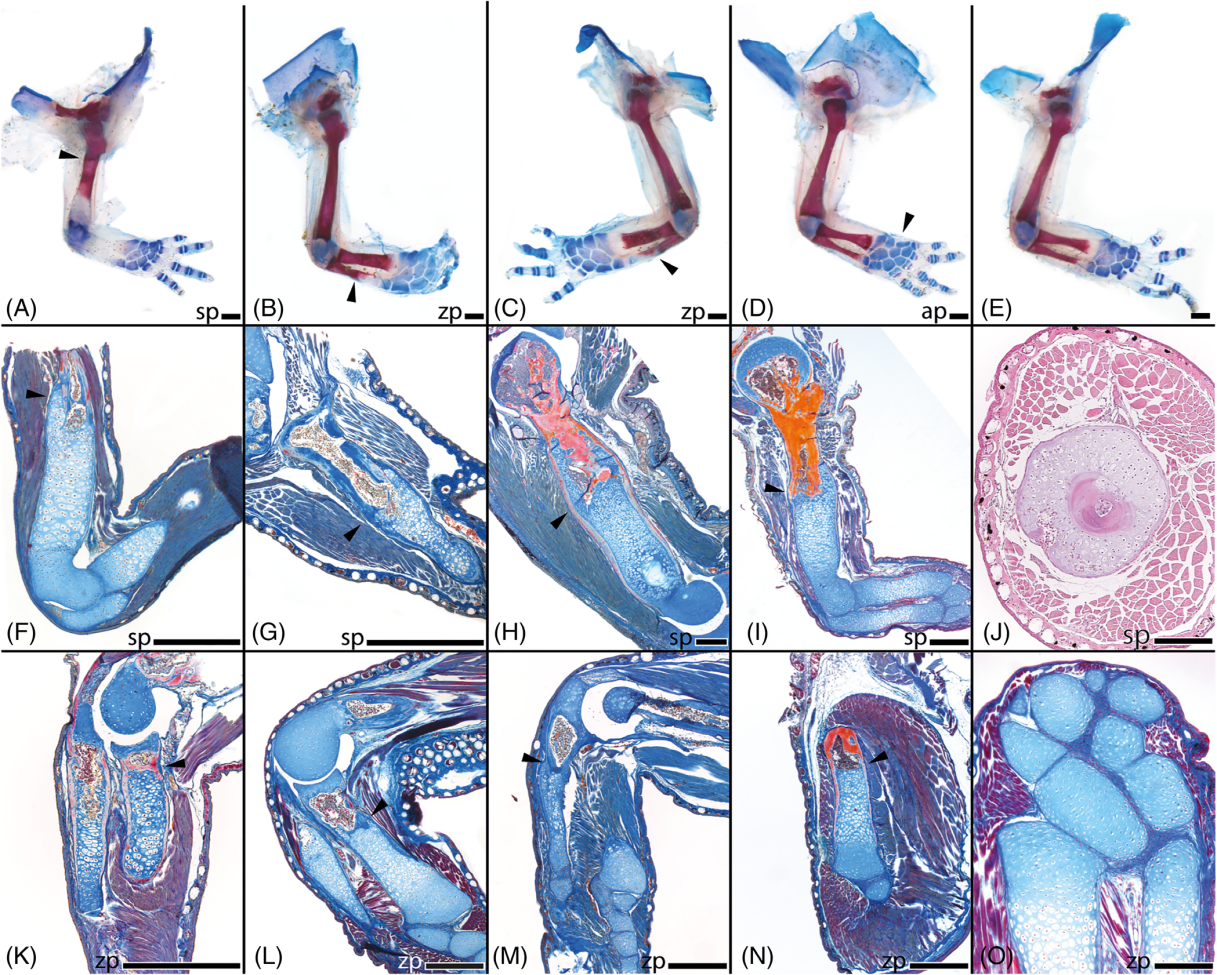
In‐lab amputations and outcomes of long‐term fore limb regeneration. Cleared and stained limbs of *D. ocoee* (A–E). Regenerated limbs (A–D). Bulky humerus (A). Bulky radius and ulna (B). Bulky radius and ulna, supernumerary carpal bone (C). Regenerated autopod without anomaly (D). Not regenerated limb, contralateral limb of 6C (E). Longitudinal histological serial sections stained with Heidenhain's Azan of regenerated limbs after controlled amputation (F–I, L–O). Amputation site humerus: *Desmognathus aeneus* RFL (F), *D. monticola* RFL (G), *D. quadramaculatus* RFL (H), *Plethodon shermani* RFL (I). Amputations site radius and ulna: *D. aeneus* RFL(K), *D. monticola* RFL (L), *D. ocoee* RFL (M), *D. monticola* LHL (N). *Desmognathus monticola* RFL, fused carpal bones (O). Transversal histological serial section stained with Hematoxylin and Eosin of *D. ocoee* RFL, bony humerus surrounded by cartilaginous callus tissue (J). RFL, right forelimb; LFL, left forelimb; LHL, left hindlimb; ap, amputation site autopod; sp, amputation site stylopod; zp, amputation site zeugopod. Black arrows highlight the amputation site. Black scale bars represent 0,5 mm.

During the regeneration process of all investigated taxa, the remaining bone stump was encased by a cartilaginous callus at the amputation site (Figures 3A–G, 5A–E,G–I, and 7F–N) forming a new robust long bone of abnormal shape (Figures 4B,C,E,F,H,I,K,L,R, 6B,C,G,J,N, and 7A–C). Histological sections stained with Heidenhain's Azan show that especially when regeneration is initiated at the humerus level, the newly formed cartilage protrudes far beyond the humerus stump (Figure 7F–I). Differences in skeletal element volumes compared to the normally developed elements were also visualized with segmented 3D models (Figure 4). These data show that regenerated long bones are larger in diameter and appear remarkably robust compared to control limbs. Interestingly, not only are the bones at the amputation site affected by this. After humeral amputation, the regenerated skeletal elements in the distal zeugopod area also appear slightly sturdier, although they retain overall normal shape (Figure 4A,B,E,H,K). In contrast, regenerated carpal bones following amputation in the cartilaginous mesopodial region (Figure 6E,H,L,O) do not appear to be more robust than the unregenerated carpal elements (Figure 6A,D,F,I,K,M), and the original amputation plane cannot be discerned after regeneration.

Pathologies occur frequently in all regions along the limb axis in regenerated limbs post amputation. Interestingly, proximal amputation can also lead to abnormalities in more distal parts of the regenerated limbs. Amputation at the stylopod level, but particularly amputations at the zeugopod and mesopod levels led to abnormalities of the autopod limb anatomy, therein most frequently to variation in the number of digits, phalanges, and carpal bones. As the mesopodium remains cartilaginous even in adult plethodontids and hence cannot be visualized in normal CT scans, staining of the soft tissue and subsequent manual segmentation was applied to examine the complete skeletal anatomy of regenerated limbs. In anatomically normal limbs of the studied species, eight carpal and nine tarsal elements are present. Following amputation at the zeugopod level in *D. aeneus*, the autopod regenerated with a supernumerary digit at the postaxial side comprised of two phalangeal elements (Figure 6B), an additional carpal element on the postaxial side positioned between distal carpal 4 and ulnare, and an enlarged ulnare (Figure 6B). In another individual of the same species, zeugopodial amputation resulted in a normal morphology of the carpus, but a reduced phalangeal formula of 1‐2‐2‐2 (Figure 6C). In one specimen of *D. monticola*, zeugopodial amputation led to a partial proximodistal amalgamation of distal carpal 4 and ulnare (Figure 7O). Amputation at the autopod level in *D. aeneus* resulted in normal carpal morphology, but the lack of the distal phalanx in digit 3 (Figure 6E). An altered phalangeal formula of 1‐2‐2‐2 was also observed in two specimens of *D. ocoee*, once after amputation at the stylopod and once at the zeugopod level (Figure 4H,I). *Desmognathus ocoee* also showed a reduced number of seven carpal bones in regenerated limbs following amputation at the zeugopod level (no image, elaborated through histological analysis). Autopodial amputation led to an incomplete regeneration in a specimen of *D. ocoee*, resulting in an autopod with only three digits (phalangeal formula 1‐2‐2, Figure 6H). Deduced from the phalangeal formula, the regenerated digits are digits I–III with one missing phalangeal element. In addition, the radiale was regenerated only rudimentarily in this individual (Figures 5F and 6H). Moreover, an additional carpal element developed in the distal region of the mesopod, possibly caused by abnormal splitting of the basale commune (combination of distal carpals 1 and 2, a unique synapomorphy of the Order Caudata) into separated carpal elements d1 and d2.

A similar pathological pattern of a regenerated hand with three digits (1‐2‐2) was also observed in a specimen of *D. quadramaculatus*, although in this case following amputation at the zeugopod. The reduced number of six carpal elements seems to be caused by multiple fusions of several carpals, both proximodistally and among laterally adjacent elements (Figures 5H and 6J). In a specimen of *P. metcalfi*, regeneration after amputation at the autopodial level yielded a limb with three digits, which are not ossified after 13 w.p.a. (Figure 2J). Due to the incomplete acropodial ossification, a conclusive clarification of the number of metacarpal and phalangeal elements was not possible, and the poor condition of the specimen prevented further investigations of the hand skeletal anatomy. Another specimen of *P. metcalfi* shows an almost anatomically correct limb regenerate following autopodial amputation, albeit final outgrowth of the digits had not been completed and ossification had not yet started after 13 w.p.a. This specimen shows only a small additional mesopodial element at the preaxial site, lateral to c1 and the basale commune (Figure 6L). Likewise, a specimen of *P. shermani* shows an almost normal limb anatomy in the regenerate following amputation at the carpal bone level, except for one notably more robust metacarpal in digit IV (Figure 6O).

### 2.2 Regeneration caused by natural appendage loss

#### 2.2.1 Gross observation of tail and limb regeneration upon collection in the wild

**FIGURE 8:**
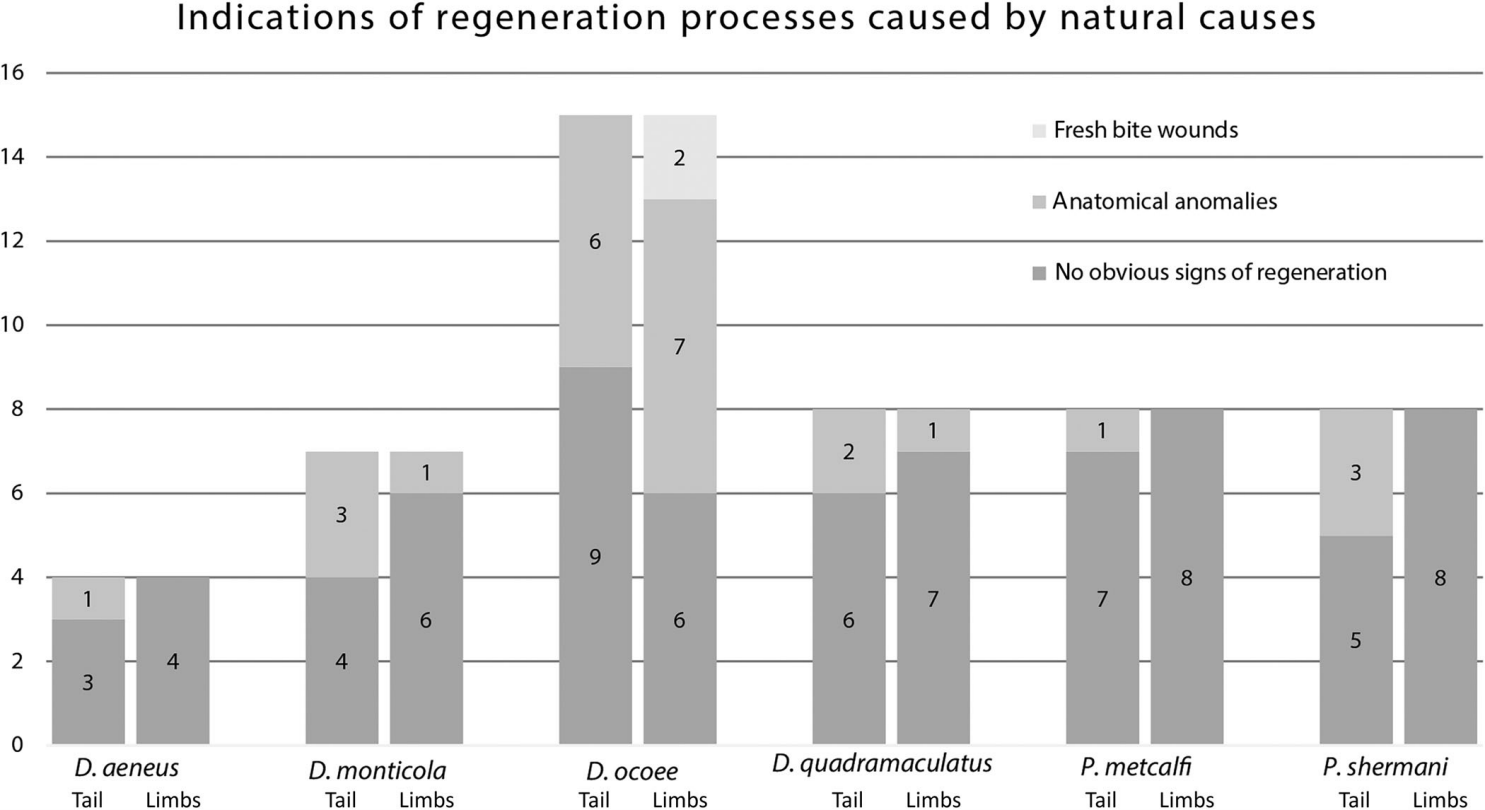
Ongoing regeneration in six plethodontid species found upon capture in the wild: *Desmognathus aeneus* (*n* = 4), *D. monticola* (*n* = 7), *D. ocoee* (*n* = 15), *D. quadramaculatus* (*n* = 8), *Plethodon metcalfi* (n = 8), and *P. shermani* (n = 8). Salamanders were investigated with respect to fresh bite wounds and anatomical anomalies of tails and limbs.

**FIGURE 9:**
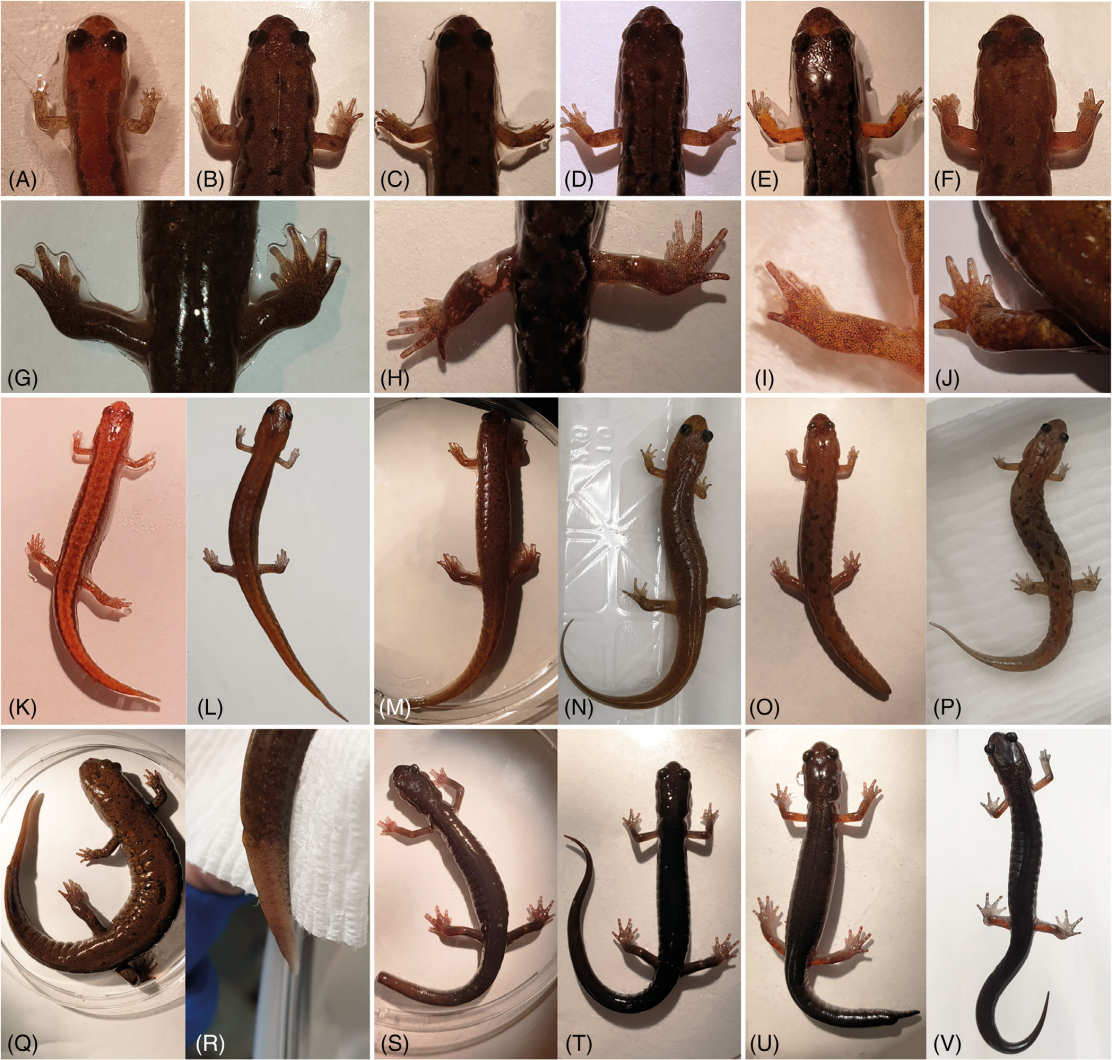
Indications of bite attacks and ongoing regeneration following natural factors at the time of collection. Forelimbs of *Desmognathus ocoee*: Thickened upper arms on the LFL (A and B), short digit I on the LFL (C), a smaller looking hand on the RFL compared to the collateral side (D), short digit I on the RFL (E), all digits shortened on the LFL (F). Hindlimbs: *D. monticola* with 4 toes on the LHL (G), *D. ocoee* with a fresh wound on the left thigh (H), *D. ocoee* with a toe with a protruding phalangeal bone on the LHL (I), *D. quadramaculatus* with a toe missing phalangeal elements (J). Tails: *D. aeneus*, ongoing tail regeneration (K), fully regenerated tail after 16 weeks (L). *Desmognathus monticola*, ongoing tail regeneration (M), fully regenerated tail after 16 weeks (N). *Desmognathus ocoee*, ongoing tail regeneration (O), fully regenerated tail after 16 weeks (P). *Desmognathus quadramaculatus*, ongoing tail regeneration (Q), fully regenerated tail after 16 weeks (R). *Plethodon metcalfi*, tail with recently severed tail (S), different individual with fully developed tail for comparison (T). *Plethodon shermani*, ongoing regeneration with evident amputation site (U), fully regenerated tail after 16 weeks (V). LFL, left forelimb; RFL, right forelimb; LHL, left hindlimb.

Upon collection in the wild, salamanders were investigated for bite wounds on the limbs and tails and, as well as any signs of ongoing regeneration and externally visible abnormalities of limb anatomy to document natural occurrences of biting and regeneration in our sample (Figure 8). Many plethodontid species are known to practice tail autotomy as an antipredator decoy strategy,^71^, ^72^, ^73^, ^74^ including the species investigated in this study. Therein autotomy involves much more applied force on the tail in plethodontids than is commonly known from lacertid lizards and may involve twisting and forceful tearing (Wake & Dresner 1967). Loss of the distal end of the tail is particularly conspicuous in the two investigated *Plethodon* species, highlighting that biting by either conspecifics and/or predators is apparently common in these populations. In one individual of *P. metcalfi*, the tail seemed to have been severed recently, because the tail was noticeably short, and ended in a plane, not a tapered end (Figure 9S) compared to the other *P. metcalfi* individuals (e.g., Figure 9T). Unfortunately, this animal died shortly after forelimb amputation, thus continuing regeneration of the tail could not be documented. In three specimens of *P. shermani*, tail regeneration had progressed to a varying extent at the time of collection, although the amputation site was still clearly discernible as the regenerating tail tip was significantly smaller in diameter than the tail stump (Figure 9U). After another 16 weeks of regeneration, the tail was significantly elongated, and the amputation site no longer evident.

Ongoing tail regeneration was not as conspicuous in any of the *Desmognathus* specimens as in the investigated *Plethodon* species. However, several specimens of *D. monticola* and *D. ocoee* showed tails that appeared overall foreshortened, but a distinct amputation site comparable to the *Plethodon* species was not identifiable (Figure 9). Nevertheless, after several more weeks in the course of the present study, the tails of these individuals were significantly longer than when the specimens were caught, strongly indicating ongoing tail regeneration (e.g., Figure 9N,P). Ongoing tail regeneration in *D. aeneus* and *D. quadramaculatus* could also be identified through comparison of tail lengths at time of capture and after 16 weeks into this study (Figure 9K,L,Q,R). However, in the specimens of these two species, differences were minimal, indicating that the final state of regeneration had almost been reached at the time of collection. Fresh bite wounds on the tails were not observed.

In terms of limb regeneration, we found several indications of previous regeneration in specimens of *D. ocoee*, the species with the largest number of samples. These included a thickened upper arms on one side (Figure 9A,B), a smaller‐looking hand compared to the co‐lateral side (Figure 9D) and forshortened digits (Figure 9C,E,F). A toe with missing phalangeal elements was observed in *D. quadramaculatus* (Figure 9J). One hind limb with four instead of five digits was identified in *D. monticola* (Figure 9G). Two specimens of *D. ocoee* showed fresh bite wounds, i.e., a flesh wound on the thigh (Figure 9H) and a toe with a protruding phalangeal bone (Figure 9I). Three of the *D. ocoee* specimens exhibited evidence for a combination of tail and limb regeneration in the form of regenerating tail tips and abnormalities in the limbs. It seems these individuals were victims of multiple bite attacks within a relatively short time span and regeneration occurred in multiple body appendages simultaneously. Interestingly, none of the direct developing species showed any anomalies or bite wounds on the limbs.

#### 2.2.2 Internal anatomy of plethodontid forelimbs

**FIGURE 10:**
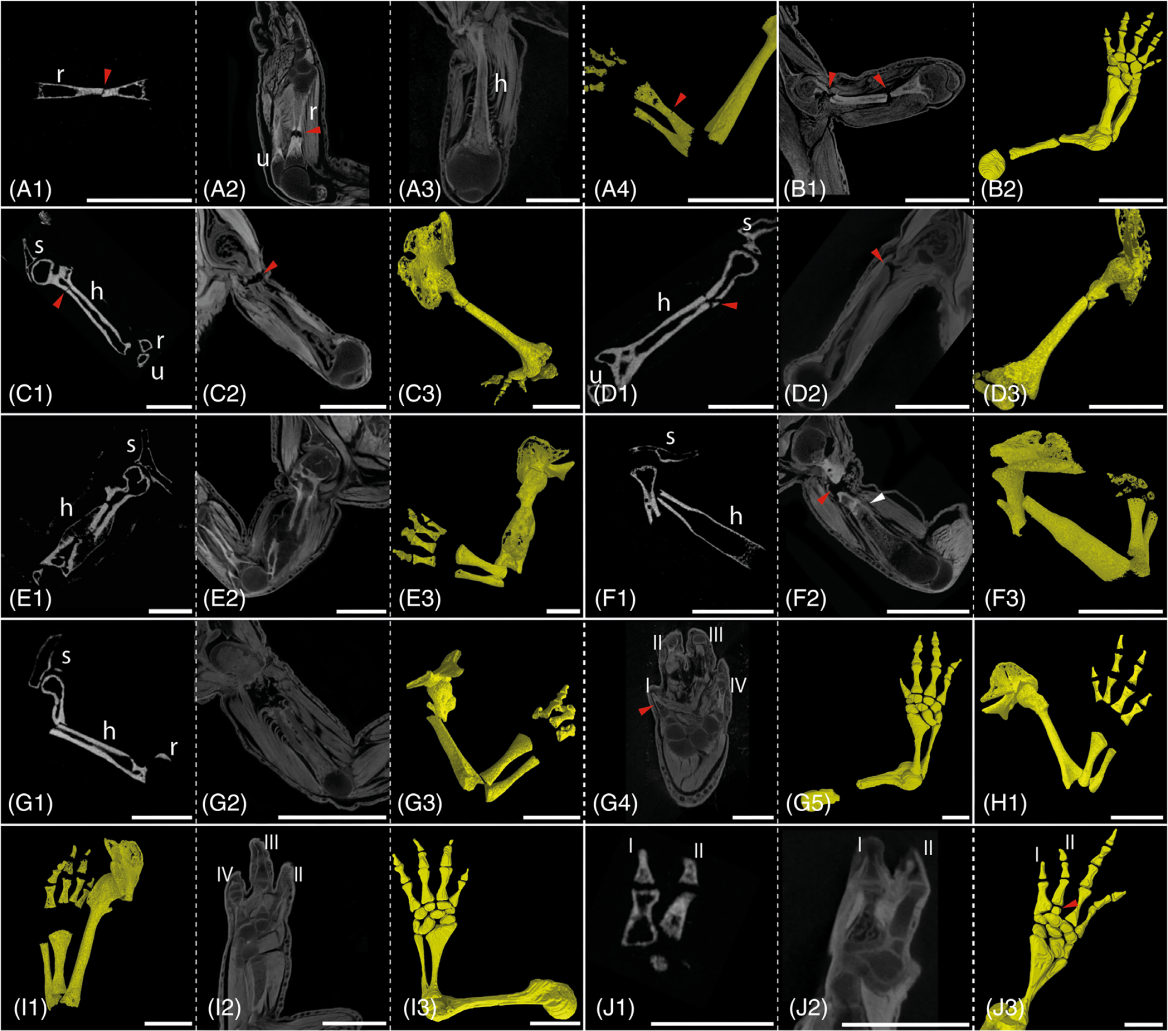
Injuries and ongoing regeneration induced by natural causes. *Desmognathus aeneus*: Fracture in stylopod (B). *Desmognathus ocoee*: Fracture in zeugopod (A). Fracture in stylopod (C–G). Missing phalanx in digit I (G). Missing phalanx in digit III (H and I). Bulky phalanx in digit I and separated phalanx in digit II (J). Micro‐CT scans unstained (A1, C1, D1, E1, F1, G1, and J1). Micro‐CT scans double stained with I2K and PTA (A2, A3, B1, C2, D2, E2, J2, G2, G4, I2, and J2). Segmented 3D models of unstained micro‐CT scans (A4, C3, D3, E3; F3, G3, H1, and I1). Segmented 3D models of stained micro‐CT scans (B2, G5, I3, and J3). h, humerus; r, radius; s, scapula; u, ulna. Red arrows indicate fractures or mis‐patterned skeletal elements. White scale bars represent 1 mm.

In addition to the externally visible injuries and/or pathologies outlined above, a number of additional abnormalities and pathologies were identified in the internal anatomy by means of CT scans and histological sections. In three limbs of *D. ocoee* an abnormal phalangeal count was identified, twice with the phalangeal formula 1‐2‐2‐2 (Figure 10H,I), once 0‐2‐2‐2 (Figure 10G). In one forelimb, anomalies were detected in the first two digits. Digit I showed a notably sturdy metacarpal, and in digit II the metacarpal was divided into a small proximal fragment and a larger distal element (Figure 10J). Mesopodial anatomy was normal in these individuals.

Non‐union fractures (failure of a fractured bone to heal) of the humerus were noted in five individuals of *D. ocoee*. Two of the humeri were broken, but not shifted in position, so that only a minimal gap could be observed between the broken parts (Figure 10C,D). The muscle fibers in one of these limbs appeared to be mostly intact, while in the other limbs they were seriously strained and partially torn (Figure 10C2). In two further individuals, the two humerus fragments were laterally misaligned (Figure 10F,G) and the muscle ruptures were severe, resulting in detachment of the muscles from the bone in some areas (Figure 10F2,G2). In none of these four specimens with fractures was evidence for initiation of a healing process via a cartilaginous matrix at the fracture site.

In one specimen with a non‐union fracture, the latter resulted in a large gap between the two fragments of the humerus (Figure 10E). In this specimen, a cartilaginous matrix encasing the ends of the bone fragments was formed to repair the broken bone. Moreover, calcified tissue had started to replace the soft callus suggesting that eventually the two bone fragments would have been rejoined if given more healing time. One individual of *D. aeneus* also showed bone fractures in the humerus in two positions (Figure 10B). Zeugopodial fracture was found in only one individual of *D. ocoee* (Figure 10A), in which the radius was broken, but the ulna remained intact. In addition to this fracture, strained musculature is visible close to the humerus in the stylopodial region of that limb (Figure 10A3).

The described fractures were not apparent in external examination and the animals seemed unperturbed by their condition, mobile, and healthy. Fractures only became apparent later in the study upon investigation via CT scans. For this reason, limb amputations were carried out as part of the amputation studies in two of these specimens with bone fractures. In the *D. aeneus* specimen, amputation was performed on the zeugopod, distal to the doubly fractured stylopod (Figure 5A). In a specimen of *D. ocoee*, amputation was performed at the humerus level, slightly distal to the fracture of that same bone (Figure 10F). Surprisingly, the already present bone fractures remained unaffected by the more distal amputation and persisted after regeneration. Both individuals regenerated a fully functional limb, albeit with a supernumerary digit in *D. aeneus* (Figure 6B), but without anatomical skeletal abnormality in *D. ocoee*.

#### 2.2.3 Internal anatomy of plethodontid hind limbs

**FIGURE 11:**
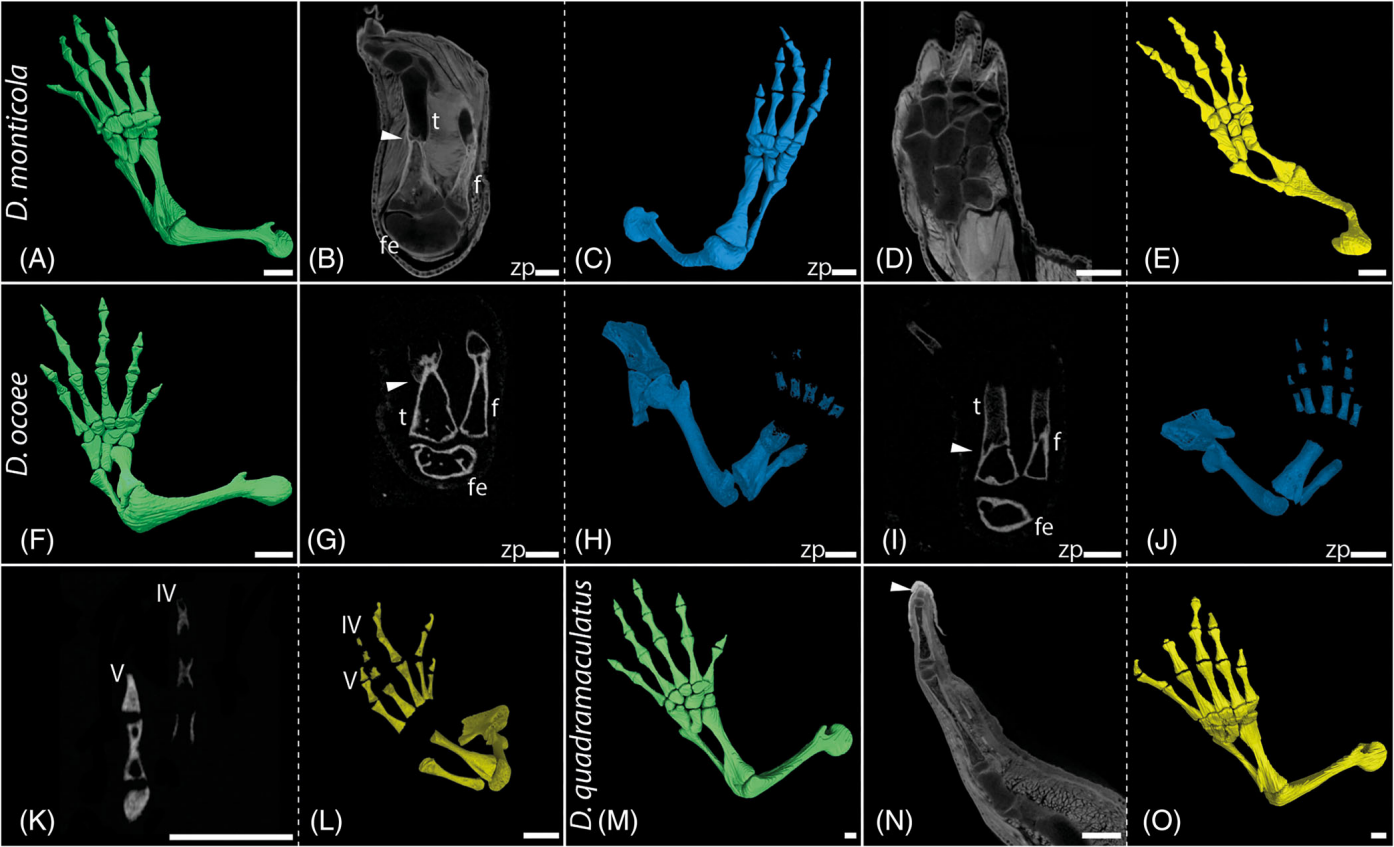
Hindlimbs of Plethodontid salamander. *Desmognathus monticola*: Representative image of normal limb anatomy (A). Following amputation at zeugopod level: Bulky tibia (B and C), missing tarsal and missing digit (C). Indication of regeneration processes caused by natural factors: Supernumerary tarsal bones (D and E) and missing digit (E). *Desmognathus ocoee*: Representative image of normal limb anatomy (F). Indication of regeneration processes caused by natural factors: Poorly ossified digit IV (G and H). Following amputation at zeugopod level: Callus encasing the bone stump (I and K). Bulky tibia and fibula (J and L) and poorly ossified digits (L). *Desmognathus quadramaculatus*: Representative image of unregenerated limb anatomy (M). Indications of regeneration processes caused by natural factors: Shorten digit IV with a vestigial phalangeal element (N) and one element completely missing (O). Micro‐CT scans unstained (I, K). Micro‐CT scans double stained with I2K and PTA (B, D, E). Segmented 3D models of unstained micro‐CT scans (H, J, L). Segmented 3D models of stained micro‐CT scans (C, F). fe, femur; f, fibula; t, tibia; zp, amputation site zeugopod. White arrows highlight the amputation site. White scale bars represent 1 mm.

The normal phalangeal formula in the hind limbs of the studied plethodontid taxa is 1‐2‐3‐3‐2 and the mesopod consists of nine tarsal elements (Figure 11A,F,M). In three specimens, a contra‐lateral amputation at the zeugopod level (tibia and fibula) was conducted to directly compare the anatomy of regenerates after naturally induced injuries and amputation, respectively. The regenerated limbs were investigated 16 weeks post amputation and compared to the limb morphology on the other side.

One individual of *D. monticola* collected in the wild exhibited only four toes on the left hind limb instead of the usual five toes (Figure 9G). X‐ray microtomographic investigation reveal the phalangeal formula 1‐2‐3‐3‐0 with digit V absent, but with supernumerary tarsal bones (Figure 11B,C) indicating an incorrect splitting of the central. The phalangeal formula corresponds exactly to that of the regenerated right hind limb after co‐lateral zeugopodial amputation in the same animal (Figure 11E). In contrast, the regenerated limb lacked one tarsal element, probably distal tarsal 5, and one digit (Figure 11E). Based on the phalangeal number of the toes, digit V was not regenerated. Moreover, one individual of *D. ocoee* was found with a protruding phalangeal bone in the left hind limb (Figure 9I). Its digit IV is completely regenerated, but the regenerated phalanges are still poorly ossified (Figure 11G,H), even after 16 weeks within this study. Histological serial sections did not reveal any change in the original number of tarsal elements. X‐ray microtomographic investigation as well as histological serial sections of the contra‐lateral regenerated hind limb after amputation showed no abnormalities in the skeletal structure.

The flesh wound (Figure 9H) on the thigh of another individual of *D. ocoee* healed completely over the course of the study. No injury or pathology to the humerus was visible after the regeneration period. Although regeneration in the contra‐lateral amputated hind limb was still in progress at the end of the study period and only metatarsals showed onset of ossification (Figure 11L), a normal phalangeal formula of 1‐2‐3‐3‐2 could be confirmed in the histological investigation. In all three cases of controlled hind limb amputation, the pattern of regeneration of the long bones is comparable to the forelimbs. The bone stump at the amputation plane is encased by a cartilaginous callus (Figure 11B,G,I, white arrows) and the regenerated tibia and fibula are sturdier than the original anatomy of the zeugopodial elements (Figure 11E,J,L).

The shortened fourth toe in an individual of *D. quadramaculatus* (Figure 9J) showed no significant elongation after 27 weeks indicating that regeneration was no longer ongoing. Micro‐CT scans showed that the abnormal digit IV consists of only the metacarpal, one normally developed phalanx and a vestigial phalangeal element (Figure 11N,O), instead of three phalangeal elements.

For some other salamander species, a substantial amount of variation in size and shape of digits, toes, and phalanges, as well as in the number and arrangements of the carpals or tarsals in unregenerated limbs has been reported.^75^, ^76^, ^77^, ^78^, ^79^ A similar amount of variation could not be identified for the specimens and taxa studied here (Figure S1). However, the restricted sample size might bias this picture and further studies with a larger number of specimens would be necessary to confirm this conclusively. The data at hand, however, clearly show that regeneration following conspecific or interspecific biting and predatory attacks occurs frequently in natural populations of plethodontid salamanders and therefore likely plays an important role for these salamander species.

## 3 DISCUSSION

Salamanders are morphologically and ecologically diverse and their highly variable life history patterns and associated phenotypic plasticity have shaped their evolution more than in any other extant tetrapod group.^80^, ^81^, ^82^, ^83^, ^84^, ^85^ Surprisingly, despite its pivotal role for salamander biology and evolution, different ecologies, habitats, and life history strategies have rarely been considered in regeneration research, which instead has thus far centered on a few model taxa, especially the axolotl. In line with this, the differences between regeneration under natural conditions and those following classical surgical amputations in laboratory settings remain poorly understood, as is the potentially adaptive role that regeneration plays in different natural salamander populations.

On the one hand, the few studies that have investigated limb regeneration in non‐model taxa, including other species of the genus *Ambystoma*,^86^, ^87^ *Bolitoglossa*,^88^ *Plethodon cinereus*,^75^, ^76^, ^89^ and *Pleurodeles waltl* ^59^, ^60^, ^90^, ^91^ reveal some variation in regeneration between these taxa and a recent study comparing limb regeneration in axolotl and newts showed that these taxa employ partially different mechanisms to rebuild tissues.^55^ This all points toward a certain degree of variation in regenerative mechanisms and patterns between different salamander taxa. On the other hand, there is some evidence from molecular as well as paleontological studies indicating a deep, shared evolutionary program of appendage regeneration in Sarcopterygians^92^, ^93^, ^94^ and possibly even Osteichthyes.^95^

In order to disentangle basic, shared aspects and programs of salamander regeneration from those that are more variable, it is crucial to acquire a broader picture and in an evolutionary context of regeneration, that includes salamander taxa representing the great ecological breadth and life history strategies. This study was taking a first step toward a better understanding of the diversity by investigating taxa that represent some of this ecological and developmental diversity.

### 3.1 Naturally induced regeneration processes

Initial observations in the field demonstrated that injuries to body appendages caused by bite wounds from conspecifics, other salamander species, and/or predators are a common phenomenon in all of the studied plethodontid species. In the three directly developing species *D. aeneus*, *P. metcalfi*, and *P. shermani*, which inhabit primarily moss and leaf litter on the forest floor, external signs of biting were found exclusively on the tails. Due to the comparatively small sample size, it remains unknown whether biting indeed focuses on the tail in these species or if the lack of ongoing limb regeneration is merely coincidental. Contrarily, bite injuries and signs of ongoing regeneration in the limbs were strikingly common in *D. ocoee*. This species shares its habitat with *D. monticola* and the preferred steep moist rock surfaces are often populated by a large number of individuals at a high density. Given that *D. monticola* has a larger body size than *D. ocoee*, it is quite conceivable that the smaller individuals become frequent victims of biting attacks in the competition for food and/or territory, and aggressive behavior was indeed frequently observed in the field. Moreover, in members of both species tail flicking behavior was observed, suggesting that tails are actively used as decoys during attacks, possibly to distract attackers from the limbs.

Specifically, flesh wounds, regenerating autopods and digits, and fractures of long bones, predominantly of the stylopod and exclusively in the forearms were observed. Bone fractures and associated muscle strains and tears are interpreted as failed biting attacks, in which the limbs were probably severely pulled and squeezed by the attacker, but amputation of the limb was not achieved. Subsequently, skin tissue and in part the musculature apparently were successfully regenerated, but healing of the fracture was not accomplished, except in a single individual. This data is partly in line with previous studies that have shown that axolotls can heal bone fractures, even in non‐fixated osteotomized bone^96^ but are apparently not capable of regenerating larger bone defects despite being capable of regenerating fully severed limbs.^97^, ^98^ Hutchison et al.^98^ demonstrated that the axolotl heals union fractures like most other vertebrates via a cartilaginous callus, but surprisingly no healing in the axolotl forelimbs with non‐union bone fractures of critical dimension (defects of a size that are large enough to preclude healing during the lifetime of the animal—CSDs^99^) was observed. Even several months after surgery, the gap length of the fracture had not changed, suggesting that the axolotl does not use the regeneration mechanisms to heal broken long bones. In contrast, except for a single case, no healing of fractures could be observed in *D. ocoee* in our study, including union‐fractures with only minimal gaps between the fragments. It is not possible to determine when the fractures were inflicted in the wild‐caught specimens, but even after a period of more than three and a half months, no indication of an ongoing healing process through cartilaginous callus formation was visible. In the study by Hutchinson et al.^98^ healing via a cartilaginous phase triggered at the fracture site and the reduction of the gap length had commenced as early as one and a half months post trauma. Reasons for the lack of fracture healing in *D. ocoee* may lie in the different preconditions of this study compared to the investigation by Hutchinson et al.^98^

The investigated specimens of *D. ocoee* were not larval as was the case in Hutchinson et al.,^98^ but sexually mature individuals that had undergone metamorphosis. It is known from axolotls that regenerative abilities of limbs decrease significantly with the age of the larvae.^41^ Moreover, Monaghan et al.^42^ demonstrated that regenerative speed in the limbs decreases significantly in axolotls, when metamorphosis is experimentally induced. This observation may indicate that metamorphosis has a strong influence on regenerative capacities in salamanders, although the decrease of regenerative speed in axolotl metamorphs may also be a direct result of the forced induction of metamorphosis, which usually does not occur in axolotls under natural conditions.

Furthermore, the investigations of Hutchison et al.^98^ focused on fractures of one of the zeugopdial elements while the other element stayed intact, which lend some support to the lower leg, prevented the limb from collapsing and preserved the distance between the detached bone fragments. In contrast, in this study fractures affected almost exclusively the humerus and hence led to lack of stability in the stylopod, which in some cases led to a shifting of the separated bone fragments.

Various explanatory hypotheses have been put forward to explain why salamanders are unable to heal non‐union bone fractures despite their impressive regeneration capabilities after amputation. The most likely factors to play a role in distinguishing bone fracture repair from regeneration after limb loss are the absence of an open wound, which is considered to be required for the successful initiation of epithelial–mesenchymal interactions during the regeneration process,^11^, ^28^, ^100^ a lack of a trauma to surrounding tissues,^11^, ^101^, ^102^, ^103^, ^104^ and the absence of dedifferentiated cells forming the blastema.^105^

It is intriguing that one individual of *D. ocoee* did show bone healing involving a calcified callus that rejoined the two bone fragments despite a large gap of critical dimension between the fragments (Figure 7J). One possible explanation, albeit speculative, could be that the injury occurred at a very early larval stage during which healing abilities might have still been higher. Considering the large dimension of the fracture, the observed fracture healing in this specimen seems to contradict the observation that all vertebrates studied to date are incapable of healing bone defects of critical dimensions.^99^ Gaps of critical dimensions are considered to be gaps between two extremities larger than a certain size where bone fractures will not heal. The interplay of fracture healing and regeneration in salamanders will have to be investigated in more detail in the future to gain a better understanding of their respective roles and limits in salamanders.

Completely bitten off limbs or serious malformations were not observed in any of the investigated specimens, although previous studies suggest that limb regeneration proceeds mostly normally after clean and controlled amputation,^106^ while malformations occur more frequently in limbs that have been bitten by conspecifics.^41^

### 3.2 Differences in the rate of regeneration between plethodontid species

All species examined in this study possess the ability to regenerate limbs after amputation without any resulting scarring or demarcation lines between the stump and the regenerated tissues. Externally, most regenerated limbs were indistinguishable from the original limb. No severe skeletal malformations in the regenerates were identified that would have significantly limited the functionality of the regenerate.

A significant correlation between life history patterns and speed of regeneration could not be identified. While *D. monticola* and *D. ocoee* seem to have completed the regeneration of the limbs after amputation at the stylopod after about 100 days, *D. aeneus* and *D. quadramaculatus* needed a longer period of time to regenerate the missing part of the limb. Regeneration in *D. aeneus* was slightly delayed with respect to final digit outgrowth despite the comparably small body size. Equivalent proportionality of replicated limbs compared to the contra‐lateral side was not achieved in *D. quadramaculatus* after 27 weeks of regeneration. This may be related to the larger body size compared to the other *Desmognathus* species. The two *Plethodon* species showed prolonged regeneration times as well. Sixteen weeks post stylopodial amputation, the autopod was not yet completely remodeled. Even after amputation at the level of the carpal bones, that is, with overall less tissue to be replicated, the digits are still significantly shortened after this regeneration time. It can be ruled out that these differences in the duration for regeneration were caused by differences in diet, temperature, or other housing settings, since all species were kept under the same conditions and fed ad libidum.

Ossification of the regenerated skeletal structures starts later in the direct‐developing species, especially in the two *Plethodon* species. While in all *Desmognathus* species, except for *D. aeneus*, the regenerated skeletal elements are completely visible in the unstained CT scans, in *P. metcalfi* and *P. shermani* the limb skeleton is still composed of cartilage with only a minimal trace of initial bone formation. Ossification commences only after the limb regenerate nearly reached its final extend. Sessions and Larson^107^ reported that there is a negative correlation between genome size and limb regeneration rate in plethodontid salamander. Although this study did not examine the same species as in this study, it indicates that *Plethodon* species generally have a larger genome than *Desmognathus* species. Therefore, our observation that *P. metcalfi* and *P. shermani* require more time to complete limb regeneration is in line with the results reported by Sessions and Larson (1987).

Nevertheless, genome size alone might not be the only factor influencing the rate of regeneration. Interestingly, salamander species that inhabit steep rock faces (*D. ocoee* and *D. monticola*) seem to regenerate faster than species that move vertically on the ground or semiaquatic species. This connection seems logical, because these climbing species strongly rely on effective locomotion and are dependent on functioning limbs to escape from predators or territorial conspecifics and for hunting prey. It is therefore possible that there is positive selection pressure on faster regeneration in climbing taxa.

In summary, while differences were detected with respect to the overall speed of regeneration and onset of ossification in regenerates in salamander taxa with different life history patterns, a clearly delineated pattern could not be identified. There appears to be a relationship between the speed of the regeneration and the habitat and different functional contexts of the limbs. However, given the comparatively small number of specimens that could be sampled for some species, further empirical data are needed to substantiate this finding and further experiments will have to investigate if regeneration under laboratory conditions differs from regeneration in the wild, especially in those species where the speed of limb regeneration seems to be influenced by their habitat. The type and extent of exposed mechanical stress during limb regeneration varies greatly between climbing salamanders inhabiting near vertical rock surfaces, terrestrial species inhabiting leaf litter and moss and aquatic or semiaquatic species where locomotion is accomplished to a large degree by lateral undulation and stroke of the tail rather than the limbs.

### 3.3 Type of pathologies

Abnormalities in regeneration occurred in all investigated species independent of the amputation plane and regardless of whether the amputation was performed in the cartilaginous or bony skeletal section. No discernible differences between species were detected in frequency, severity, or type of malformation, which can be subdivided into two major categories.

First, bulky long bones in stylopod, zeugopod, and metacarpal elements of regenerated limbs occurred in all studied species, except for *P. metcalfi*. However, the lack in the latter species is likely due to the paucity of data and the late onset of ossification in this species. Surprisingly, unusually robust regenerated bony elements did not occur exclusively in immediate proximity to the area of amputation. Whereas zeugopodial amputation led to abnormally shaped radius and ulna, and autopodial amputation produced thickened metacarpals, stylopodial amputation, in contrast, produced more robust and abnormal shapes in the humerus as well as in the distal elements radius and ulna. The same observation was recently made by Kaucka et al.,^60^ who revealed fundamental differences between regeneration and development of the limb skeleton. Using the axolotl, both as larval form and after artificially induced metamorphosis, as well as the aquatic *P. waltl*, they showed that altered developmental programs and oriented cell divisions cause long bone regenerates with increased diameter compared to normal bones in these salamanders. Contrary to developmental limb growth, skeletal regeneration is characterized by uncoupled, successive chondrogenesis and ossification. Cartilage expansion occurs until the limb is reaching its final size and shape. The late onset of ossification starting from the cortical bone and proceeding inwards is not restricting the transversal cartilage expansion, resulting in bulkier skeletal elements.^60^ Possibly, these robust limb skeletal elements provide more stability for weight bearing during the regeneration process and ensure a fully functional limb thereafter.^60^

Abnormalities in autopodial elements are also frequent after regeneration, especially following amputation at the wrist. We noted a substantial deviation in size, shape, quantity, and arrangement of carpal or tarsal elements, as well as variation in the number of digits and phalangeal elements. Reduction of mesopodial and phalangeal elements in salamanders after regeneration result from either the failure of an element to form at all during regeneration, formation of an element but failure to segment, or an amalgamation of neighboring elements. Supernumerary skeletal elements emerge through extended sequences of segmentation or bifurcation.^78^ Whether these incorrectly replicated limbs with unusual mesopodial patterns may be related to the mechanical factors impinging on the regenerating limb or to external or internal environmental factors remains unknown.

Frequent and extensive intraspecific variability of autopod skeletal anatomy including different combinations of fused adjacent mesopodial elements and variant phalangeal patterns is known from many wild populations of different salamander species including *Triturus*,^77^ *Taricha granulosa*,^78^ or *Plethodon cinereus*.^75^, ^76^, ^89^ However, the observed regenerative anomalies are distinct from extensive native intrapopulation variation in limb‐skeletal patterning during initial limb development and differ qualitatively and quantitatively from them.^92^ This is confirmed in this study, where carpal bones of unamputated forelimbs served as control for intraspecific variation. While shape and arrangement of carpal bones in the species studied here may vary slightly in some cases, the absolute number of carpal bones did not vary.

### 3.4 Why do some individuals show pathologies and others do not?

There are several possibilities to explain the diversity of the described anomalies in regenerates and why some individuals show pathologies in the limbs after amputation and others do not.

On the one hand, it has been demonstrated that age is negatively correlated with regenerative capacities in salamanders.^41^ Although axolotls are able to regenerate throughout their lifespan,^108^ even larvae exhibit different regeneration abilities according to their developmental stage.^41^ In very young larval stages, limb regeneration seems to be better buffered against abnormalities in regeneration, resulting in a high frequency of perfectly regenerated limbs. As the larvae get older, limb regeneration still proceeds, but is evidently more prone to malformations. In metamorphosed axolotl, the regeneration rate decreases significantly, and pathologies occur more frequently.^42^ However, age determination in salamanders collected in the wild cannot easily be done beyond the basic developmental stages (hatchling, larva, metamorph, and adult). Skeletochronology, a technique used to determine the ages of amphibians by counting lines of arrested growth (LAGs) is controversial and often leads to inaccurate results.^109^, ^110^, ^111^, ^112^

Furthermore, it has been documented that repeated limb amputations result in abnormalities in the limb anatomy including gross morphological deviations from normal limb size and shape or alterations in the normal arrangement of skeletal elements.^9^, ^113^, ^114^, ^115^ There is the possibility that some of the individuals were victims of natural bite injuries followed by full regeneration prior to collection for the present study. In these individuals, conducted amputations might indeed repeat regeneration processes, resulting in pathologies.

Dinsmore and Hanken^89^ reported that in *P. cinereus*, amputation level has a significant effect on the incidence of specific mesopodial fusion patterns of carpal and tarsal bones in the regenerated limbs. Stylopodial amputation leads to mesopodial patterns with predominantly preaxial fusion combinations, whereas postaxial fusion combinations dominate in zeugopodial amputations.

## 4 CONCLUSIONS

The data presented in this study provide insights into the diversity of regenerative processes in different salamander species and the potential influence of life history, habitat and locomotion on the speed and accuracy of limb regeneration. The enormous diversity of salamanders in the Appalachian Mountains representing all life history strategies, many different habitats and ecologies provides a unique opportunity to start to disentangle the influence of these factors on regenerative processes. Plethodontid salamanders are well‐suited non‐model taxa for these questions in regeneration. Naturally, studies based on wild‐caught species come with several additional considerations, including collection restrictions, the impact on populations, seasonal availability of specimens, and non‐controllable parameters such as age determination and previous regeneration events. Nevertheless, results from non‐model species can supplement the vast amount of data generated based on model taxa. They contribute new insights into the diversity of mechanisms involved in salamander limb regeneration and are essential for disentangling shared versus derived evolutionary patterns of limb regeneration in salamanders specifically and tetrapods as a whole. This evolutionary perspective provides a novel framework for studies on the morphological and molecular basis of limb regeneration and highlights new research avenues.

However, working with non‐model taxa is challenging, especially with taxa collected in the field, as this approach limits the number of specimens available for study and aspects such as age, sex, or possible previous regenerations cannot be well constrained. Therefore, studies on natural populations and non‐model taxa can complement, but certainly not replace studies on model organisms.

## 5 EXPERIMENTAL PROCEDEURES

In order to maximize the data gained from the available material, a variety of methods was applied, including a combination of micro‐CT imaging techniques, clearing and staining techniques, and histological serial sections. See Table S1 for details on which techniques were applied to particular specimens.

### 5.1 Ethics statement

The collection of the animals was authorized by the North Carolina Wildlife Resources Commission (Wildlife Collection License—Permit number 15‐SC01004/19‐SC01338/Endangered Species Permit—19‐ES00569). Experimental procedures were carried out in strict accordance with the guidelines for animal care and husbandry and approved by the Institutional Animal Care and Use Committee of Highlands Biological Station (North Carolina). Animal husbandry standardized methods were performed according to the University of Chicago ACUP (Animal care and use protocol). Animals were anesthetized and euthanized with tricaine methanesulfonate (MS‐222, Sigma) and efforts were made to minimize suffering.

### 5.2 Animals

Adult specimens of six species of plethodontid salamanders were collected, including four of the genus *Desmognathus* and two of the genus *Plethodon*. Due to permit restrictions and collection success, the quantity of samples per species differs. The species vary in terms of life history patterns and habitats (for overview see Table 1).

### 5.3 Field and gross observations

The density of individuals in favorable habitats can be extremely high at the collection sites and several species of different sizes may inhabit the same area. Biting and attacks by conspecifics and individuals of other species are very frequent at these localities and can be observed directly. Salamanders were checked for bite wounds and obvious signs of ongoing regeneration. In order to detect completed regeneration, particular attention was paid to anomalies deviating from the original external skeletal anatomy.

### 5.4 Amputations

Salamanders were anesthetized with tricaine (MS 222, Sigma; 0.1% for small individuals, 0.2% for larger individuals), and the limbs were amputated using micro scissors (Fine Science Tools) at defined levels along the proximo‐distal limb axis (stylopod, zeugopod, and carpal/tarsal level). The right forelimb was used for surgery, except in cases of a suspected previous regeneration. In those specimens the left limb was amputated. After amputations, animals were rinsed with water to remove traces of tricaine and were returned to plastic containers with moist, clean paper towel for recovery.

### 5.5 Husbandry

Animals were housed in plastic containers (15 × 22 × 10 cm) padded with clean paper towel soaked with spring water. Animals were provided with hiding places and kept in a climate chamber at 18°C with a 12 h light–12 h dark cycle. Salamanders were examined daily. Depending on the size of the animals, they were fed flightless fruit flies (*Drosophila melanogaster*), mealworms, earthworms, and/or crickets three times per week. After a defined timespan (114d/*D. aeneus*, 118d/*D. monticola* and *D. ocoee*, 192d/*D. quadramaculatus*, 116d/*P. metcalfi* and *P. shermani*), animals were euthanized with tricaine (3% for smaller individuals, 4% for larger individuals) and fixed in 4% formalin for about 48 hours before transfer to 70% ethanol.

Not all of the surgically treated salamanders survived the entire duration of this study (*D. monticola n* = 2Ɨ, *D. quadramaculatus n* = 3Ɨ, *P. metcalfi n* = 5Ɨ, *P. shermani n* = 4Ɨ). Especially the *Plethodon* species seemed to be very sensitive to either the operations themselves or housing in the laboratory. This loss decreased the sample size of some species significantly, restricting broader intraspecific comparisons.

### 5.6 Computed tomography/3D reconstructions

Micro‐tomographic analyses were performed with a Phoenix nanotom X‐ray|s tube. Scan parameters were adapted for each specimen (Table S1). The cone beam reconstruction was performed using the datos|x‐reconstruction software (GE Sensing & Inspection Technologies GMBH phoenix|x‐ray datos|x 2) and data were visualized in VG Studio Max 3.5.2. Unstained scans were automatically segmented, but stained Ct scans required manual segmentation. For stained CT scans regenerated and unregenerated limbs were removed from the body and stained for 1 week each with 1% iodine in sterile distilled water and 1.5% PTA in sterile distilled water (increasing concentration to prevent shrinking).

### 5.7 Histology

Serial histological sections were prepared from selected samples following standard protocol^116^ and stained with Heidenhain's Azan or hematoxylin and eosin (see Table S1). Sections were analyzed and documented using the Leica DFC495 Digital Color Microscope Camera mounted on a Zeiss Axioskop and the Leica Application Suite V 4.2. Software.

### 5.8 Clearing and staining

Clearing and staining followed standard procedures after Ovchinnikov (2009) with minor modifications. Therein, specimens were skinned, stained in a 0.015% Alcian‐blue‐solution for approximately 12 h and washed afterwards in an ethanol series. Maceration was performed in trypsin (0.1%, Sigma‐Aldrich) for 1 week at 37°C. Ossified skeletal elements were stained with 0.01%‐Alizarin‐red‐solution for approximately 6 h and washed afterward in a 30%‐glycerin solution. For final storage, the samples were transferred to glycerin.

## Supporting information


**Figure S1.** Autopod anatomy of cleared and stained unregenerated limbs. *D. aeneus*, RFL (A). *D. monticola*, RFL (B). *D. ocoee*, RFL (C), LFL (D1‐4), RHL (D5‐6). *D. quadramaculatus*, RFL (E). *P. metcalfi*, RFL (F). *P. shermani*, RFL (G). RFL = right forelimb, LFL = left forelimb, RHL = right hindlimb. Dotted lines represent phalanges that were accidentally detached during preparation. Black scale bars represent 0,5 mm.

**Table S1.** Detailed overview of methods applied to each particular specimen.
