# Supplementary material for: Effects of life history strategies and habitats on limb regeneration in plethodontid salamanders
Source: Dev Dyn. 2024 Sep 20;254(5):396–419. doi: 10.1002/dvdy.742 (PMC12047434; doi:10.1002/dvdy.742)
Supplement: Supplementary file 1 — Figure S1. Autopod anatomy of cleared and stained unregenerated limbs. D. aeneus, RFL (A). D. monticola, RFL (B). D. ocoee, RFL (C), LFL (D1‐4), RHL (D5‐6). D. quadramaculatus, RFL (E). P. metcalfi, RFL (F). P. shermani, RFL (G). RFL = right forelimb, LFL = left forelimb, RHL = right hindlimb. Dotted lines represent phalanges that were accidentally detached during preparation. Black scale bars represent 0,5 mm. [file DVDY-254-396-s001.docx]

**
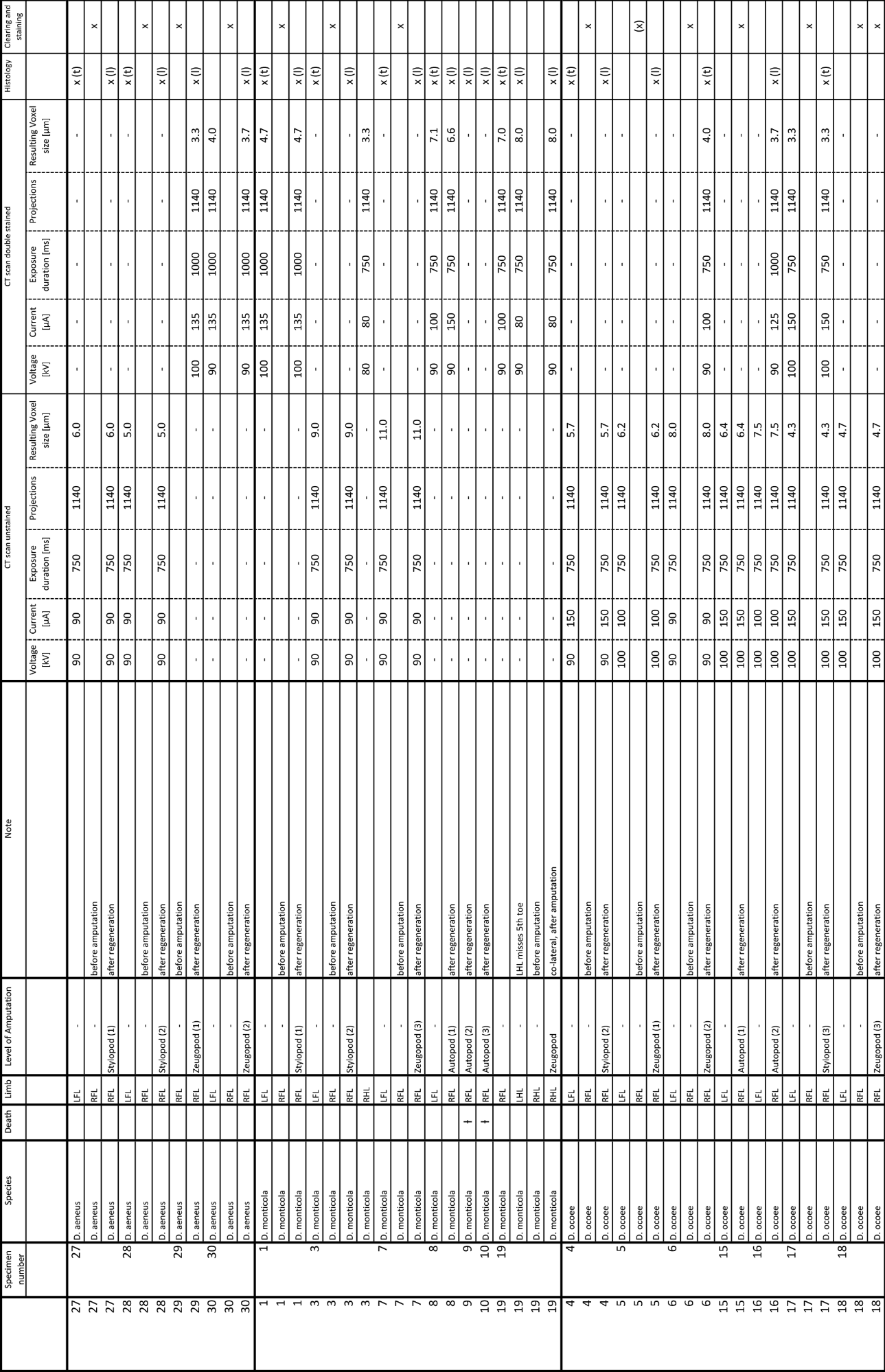
Table 1:** Detailed overview of methods applied to each particular specimen

**
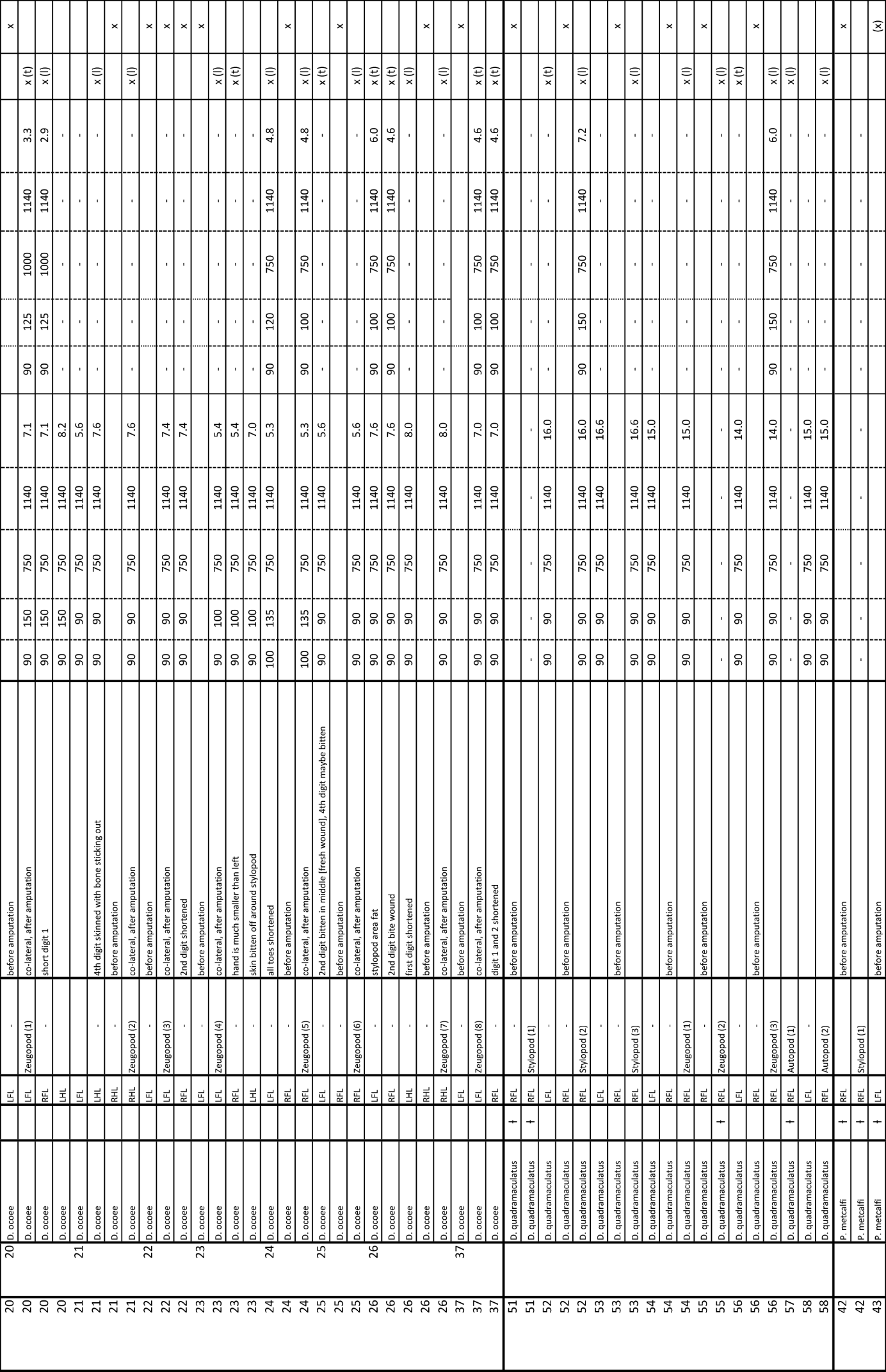
**

**
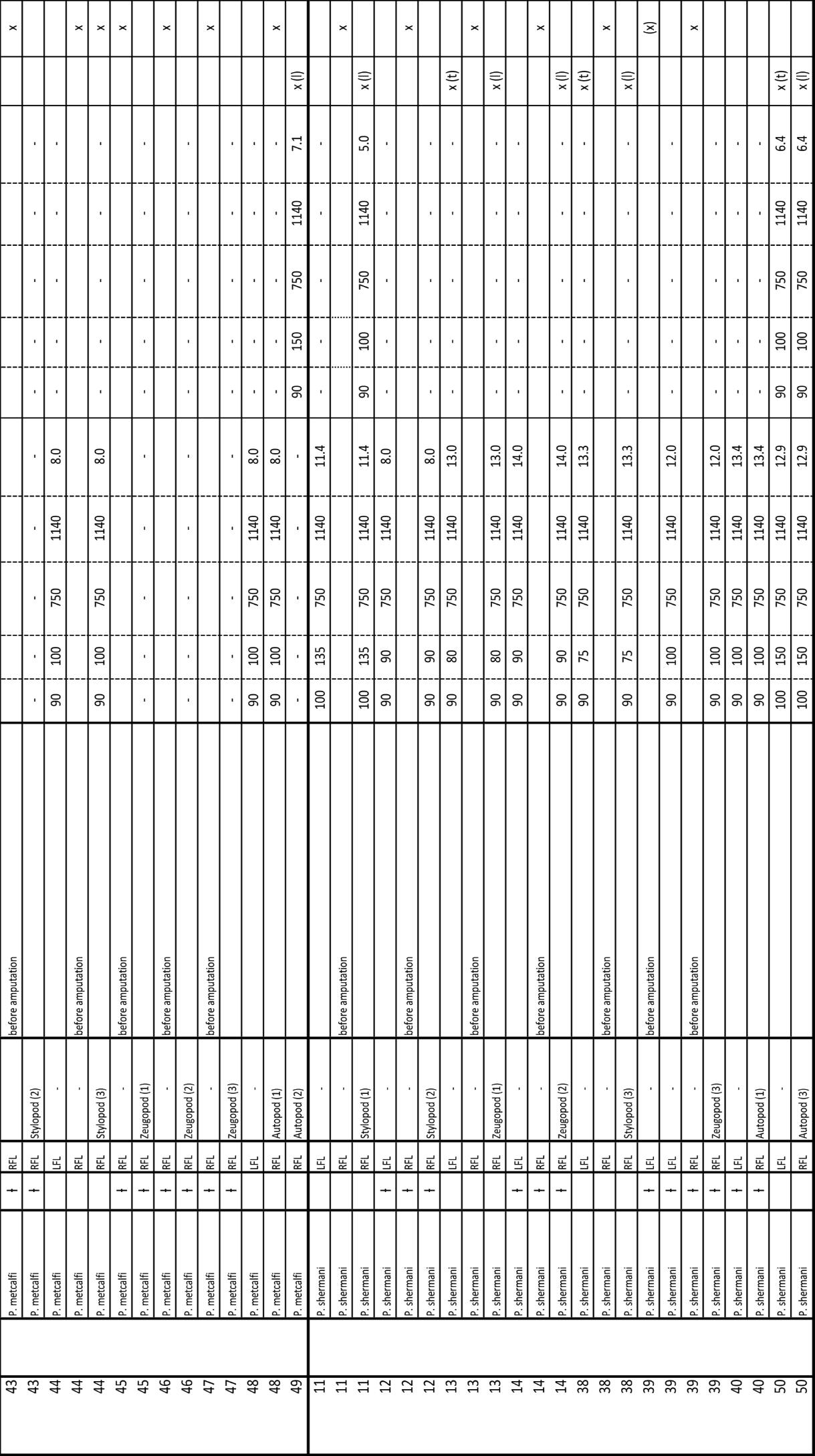
**

**
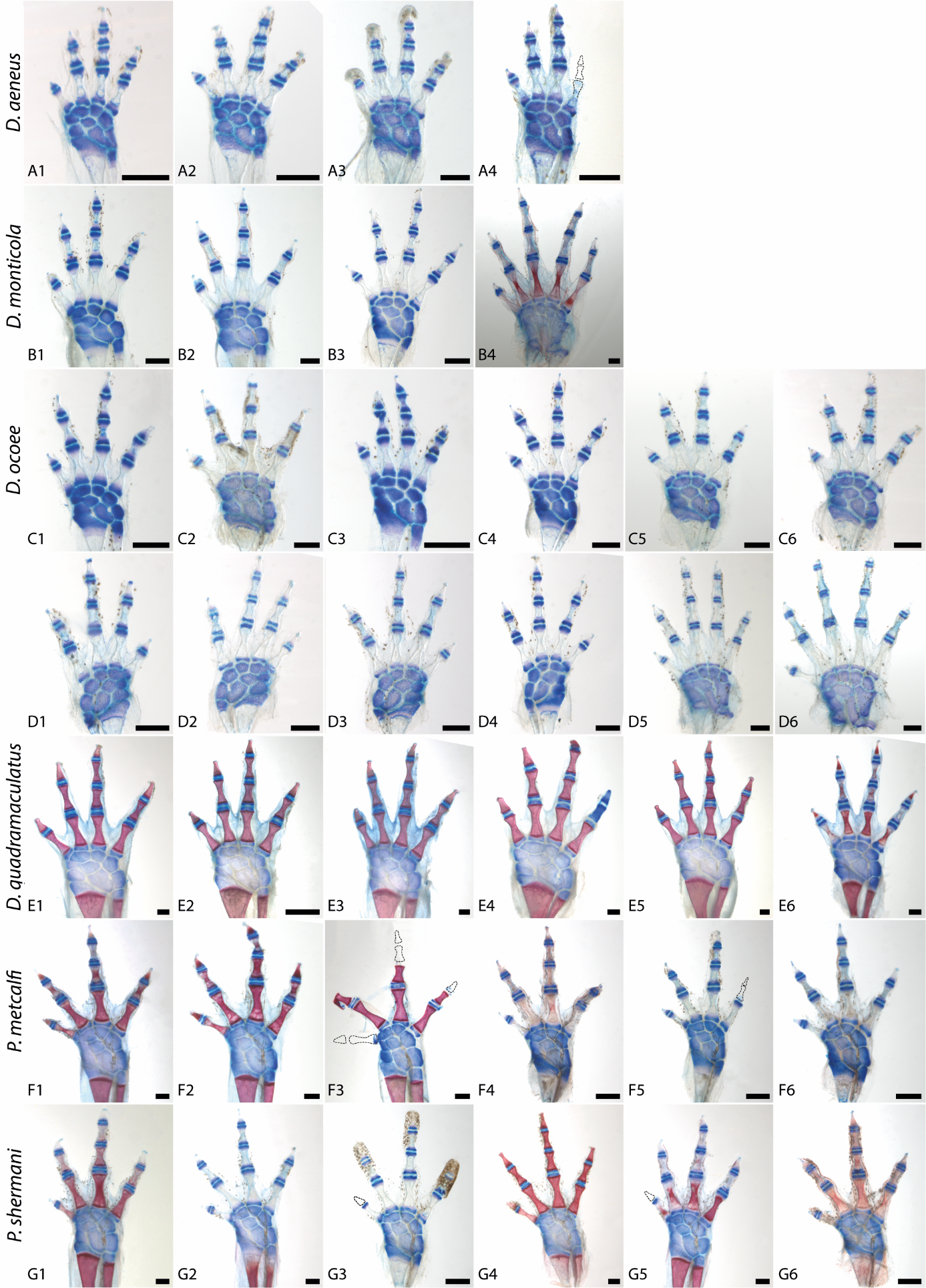
**

**Figure 1:** Autopod anatomy of cleared and stained unregenerated limbs. *D. aeneus*, RFL (A). *D. monticola*, RFL (B). *D. ocoee*, RFL (C), LFL (D1-4), RHL (D5-6). *D. quadramaculatus*, RFL (E). *P. metcalfi*, RFL (F). *P. shermani*, RFL (G). RFL = right forelimb, LFL = left forelimb, RHL = right hindlimb. Dotted lines represent phalanges that were accidentally detached during preparation. Black scale bars represent 0,5mm.
